# Weighted Gene Co-Expression Network Analysis Identifies Key Modules and Central Genes Associated With Bovine Subcutaneous Adipose Tissue

**DOI:** 10.3389/fvets.2022.914848

**Published:** 2022-06-22

**Authors:** Hui Sheng, Cuili Pan, Shuzhe Wang, Chaoyun Yang, Junxing Zhang, Chunli Hu, Honghong Hu, Xue Feng, Mengli Yang, Zhaoxiong Lei, Yuhong Gao, Zhong Wang, Yun Ma

**Affiliations:** ^1^Key Laboratory of Ruminant Molecular and Cellular Breeding, School of Agriculture, Ningxia University, Yinchuan, China; ^2^College of Life Sciences, Xinyang Normal University, Xinyang, China

**Keywords:** fat deposition, WGCNA, Hub gene, focal adhesion, PI3K-AKT

## Abstract

**Background:**

Fat deposition is an important economic trait in livestock and poultry production. However, the relationship between various genes and signal pathways of fat deposition is still unclear to a large extent. The purpose of this study is to analyze the potential molecular targets and related molecular pathways in bovine subcutaneous adipose tissue.

**Results:**

We downloaded the GSE116775 microarray dataset from Gene Expression Omnibus (GEO). The weighted gene co-expression network (WGCNA) was used to analyze the gene expression profile, and the key gene modules with the highest correlation with subcutaneous adipose tissue were identified, and the functional enrichment of the key modules was analyzed. Then, the “real” Hub gene was screened by in-module analysis and protein–protein interaction network (PPI), and its expression level in tissue samples and adipocytes was verified. The study showed that a total of nine co-expression modules were identified, and the number of genes in these modules ranged from 101 to 1,509. Among them, the blue module is most closely related to subcutaneous adipose tissue, containing 1,387 genes. These genes were significantly enriched in 10 gene ontologies including extracellular matrix organization, biological adhesion, and collagen metabolic process, and were mainly involved in pathways including ECM-receptor interaction, focal adhesion, cAMP signaling pathway, PI3K-AKT signaling pathway, and regulation of lipolysis in adipocytes. In the PPI network and coexpression network, five genes (CAV1, ITGA5, COL5A1, ABL1, and HSPG2) were identified as “real” Hub genes. Analysis of Hub gene expression by dataset revealed that the expression of these Hub genes was significantly higher in subcutaneous adipose tissue than in other tissues. In addition, real-time fluorescence quantitative PCR (qRT-PCR) analysis based on tissue samples and adipocytes also confirmed the above results.

**Conclusion:**

In this study, five key genes related to subcutaneous adipose tissue were discovered, which laid a foundation for further study of the molecular regulation mechanism of subcutaneous adipose tissue development and adipose deposition.

## Introduction

Adipose tissue is an active metabolic organ that secretes numerous protein factors, such as resistin and lipocalin, which are essential for lipid accumulation, energy expenditure, glucose, and insulin metabolism, and hormonal regulation, and have a profound role in regulating the body's glucolipid metabolic homeostasis and maintaining energy homeostasis (1, 2). However, due to the complex molecular mechanism of adipose tissue formation and deposition, there are relatively few studies on adipose deposition-related genes in cattle, which need to be further explored.

Adipose tissue not only plays a necessary role in the development of individuals but also plays an important role in the study of beef quality. Adipose tissue is classified as intramuscular fat, visceral fat, intermuscular fat, and subcutaneous fat according to its distribution. Among them, intramuscular fat (IMF) plays an indispensable role in meat quality, which affects meat taste, juicy, shear force, and so on (3, 4). Subcutaneous adipose tissue is an important source of essential fatty acids and plays a role in the transport of fat-soluble vitamins, which constitute the main source of energy and insulating substances for the animal's body. Studies have shown that fat deposition in backfat is related to body fat percentage, carcass cross-sectional fat area ratio, intramuscular fat, and flavor and juiciness of beef (5, 6). In Japanese Wagyu cattle, IMF content in the longest dorsal muscle was found to be positively correlated with the percentage of subcutaneous fat and maturity *in vivo* (7, 8). Therefore, the identification of key genes related to subcutaneous adipose tissue by a new method is an important strategy for carcass and meat quality genetic improvement.

Weighted gene coexpression network analysis (WGCNA) is an efficient and accurate method for bioinformatics and biological data mining (9). It helps to create free-scale gene co-expression networks to identify associations between different gene collections or between gene collections and clinical features (10). At present, it is widely used in genetics, cancer, and brain imaging research and analysis (11). By using WGCNA, we can create a coexpression network to identify differentially related gene clusters and analyze gene specificity (12).

This study constructed a gene co-expression network using transcriptome data from multiple tissues and identified the modules of gene co-expression related to subcutaneous adipose tissue. The interested module was analyzed by gene ontology (GO) and Kyoto Encyclopedia of Genes and Genomes (KEGG) pathway analysis, and the Hub gene in the module was determined. Then the expression of the Hub gene was analyzed, and the accuracy of selection was preliminarily verified. This study provides a starting point for further exploration of the molecular regulatory mechanism of fat deposition.

## Materials and Methods

### Data Collection and Preprocessing

We downloaded the GSE116775 microarray dataset from the Gene Expression Omnibus (GEO) database (Supplementary Table 1), which consists of approximately 1.2 Tb of high-quality RNA-Seq transcriptome data from four key metabolic tissues (rumen epithelium, liver, muscle, and subcutaneous fat) and data on the content of 49 fatty acids in backfat, to explore the molecular regulatory mechanisms of these tissues in fatty acid formation in cattle (13). In our study, the GSE116775 dataset was used to construct co-expression networks and identify hub genes associated with bovine subcutaneous adipose tissue. The data set provides gene expression profiles of 185 samples containing four tissue types of three breeds of cattle. All the datasets were normalized independently using Robust Multiarray Average (RMA) followed by log2 transformation and quantile normalization.

### Construction of Co-Expression Network

Phenotype-correlated gene modules associated with adiposity were identified by WGCNA. The top 10,000 genes with the highest expression levels were used to construct the WGCNA network using the WGCNA package in R. Firstly, a similarity matrix was constructed by calculating the Pearson's correlation coefficient to measure the similarity between all samples. Then, based on the free-scale topological criterion, the similarity matrix is transformed into an adjacency matrix, and a scale-free co-expression network based on the soft threshold parameter β is constructed by using the adjacency matrix. In this study, the free-scale topology was R^2^ = 0.80 with a soft threshold equal to 12. The topological overlap matrix (TOM) was used to define modules based on dissimilarity (1-TOM). The minimum number of genes in each module is 30. For module grouping, the partition threshold used is 0.25. Finally, a color is assigned to each gene module, and genes that are not assembled into any module are grouped into gray modules (14).

### Identification of the Module of Interest and Functional Annotation

In order to clarify the potential biological significance of genes in key modules and further explore the functions of genes in the most related modules of subcutaneous adipose tissue, we analyzed GO terms and KEGG pathways using the “clusterProfiler” software package in R software. In addition, only when the p.adjust of the GO or KEGG terms is >0.01, they are considered important.

### Hub Genes Identification

The degree of module members (MM) is defined as the correlation between gene expression profiles and module eigengenes (Mes). Gene significance (GS) is defined as the absolute value of the correlation between genes and external traits. Genes with both high MM and high GS are considered to be candidate central genes. Then, all the genes in the interested module are uploaded to the STRING website to create a PPI network, and the CytoHubba plug-in of Cytoscape software is used to identify the central genes in the PPI network. Hub genes that co-occurred in the co-expression network and Cytoscape software analysis were considered as “real” Hub genes and were selected for subsequent analysis (15, 16).

### Validation of the Hub Genes

The expression levels of Hub genes in the dataset were analyzed by differential expression patterns. At the same time, qRT-PCR was used to further verify the expression level of Hub gene in bovine subcutaneous adipose tissue and bovine preadipocytes at different induction stages. The R package ggpubr was used to calculate difference significance and visualization.

### Sample Collection and Cell Culture

The tissue samples (rumen epithelium, liver, muscle, and subcutaneous fat) and bovine subcutaneous adipocytes were provided by the Key Laboratory of Ruminant Molecular Cell breeding of Ningxia University. All the experiments were carried out in strict accordance with the recommendations in the guidelines for Animal Protection and Utilization of Ningxia University and approved by the Animal Welfare Committee of Ningxia University (IACUC-NXU1098). The isolated cells were cultured in a growth medium containing 90% Dulbecco's modified Eagle medium (DMEM, Gibco) and 10% fetal bovine serum (FBS, Gibco) until cell density reached approximately 80%. Then 90% DMEM, 10% FBS, 3.5 mg/ml insulin (Sigma), 0.01% dexamethasone (Sigma), 1% Isobutylmethylxanthine (Sigma) and 0.01% rosiglitazone (Sigma) were mixed together to form a differentiation medium to induce cell differentiation. After 2 days of differentiation, the cells were cultured in maintenance medium (95% DMEM, 5% FBS, 3.5 mg/mL insulin, 0.01% rosiglitazone).

### Oil Red O Staining

Oil red O was used to observe the morphology of bovine preadipocytes after 0 and after 10 days of differentiation. The storage solution of oil red O (oil red O + isopropanol) was diluted into a working solution at 2:3 with Phosphate-Buffered Saline (PBS), and the cells were sfixed with 10% formalin and stained (17).

### RNA Extraction and qRT-PCR

Total RNA was extracted from tissue samples and cultured cells using TRIZOL reagent (Invitgen, USA). PrimeScript II first strand cDNA synthesis kit (Takara, Dalian, China) was used to prepare first strand cDNA. QRT-PCR was performed using All-in-One™ qRT-PCR Mix (Genocopoeia, Guangzhou, China) in a LightCycler^®^ 96 Instrument (Roche, Germany) to detect the expression level of mRNAs. All the primers used are listed in Supplementary Table 2.

### Statistical Analysis

According to the characteristics of data distribution, a nonparametric test or t-test was used to analyze the statistical significance of gene expression in four tissues. Tissue and cellular qRT-PCR experiments were performed using mRNA of β-actin as an endogenous control at basal levels, and the relative expression levels of genes were calculated using the 2^−ΔΔCt^ method and were considered statistically significant when the *p* < 0.05.

## Results

### Construction of Weighted Gene Co-Expression Network

**Figure 1:**
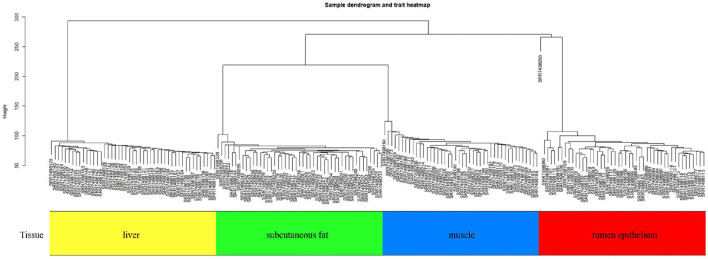
Sample dendrogram and trait heatmap in GSE116775.

**Figure 2:**
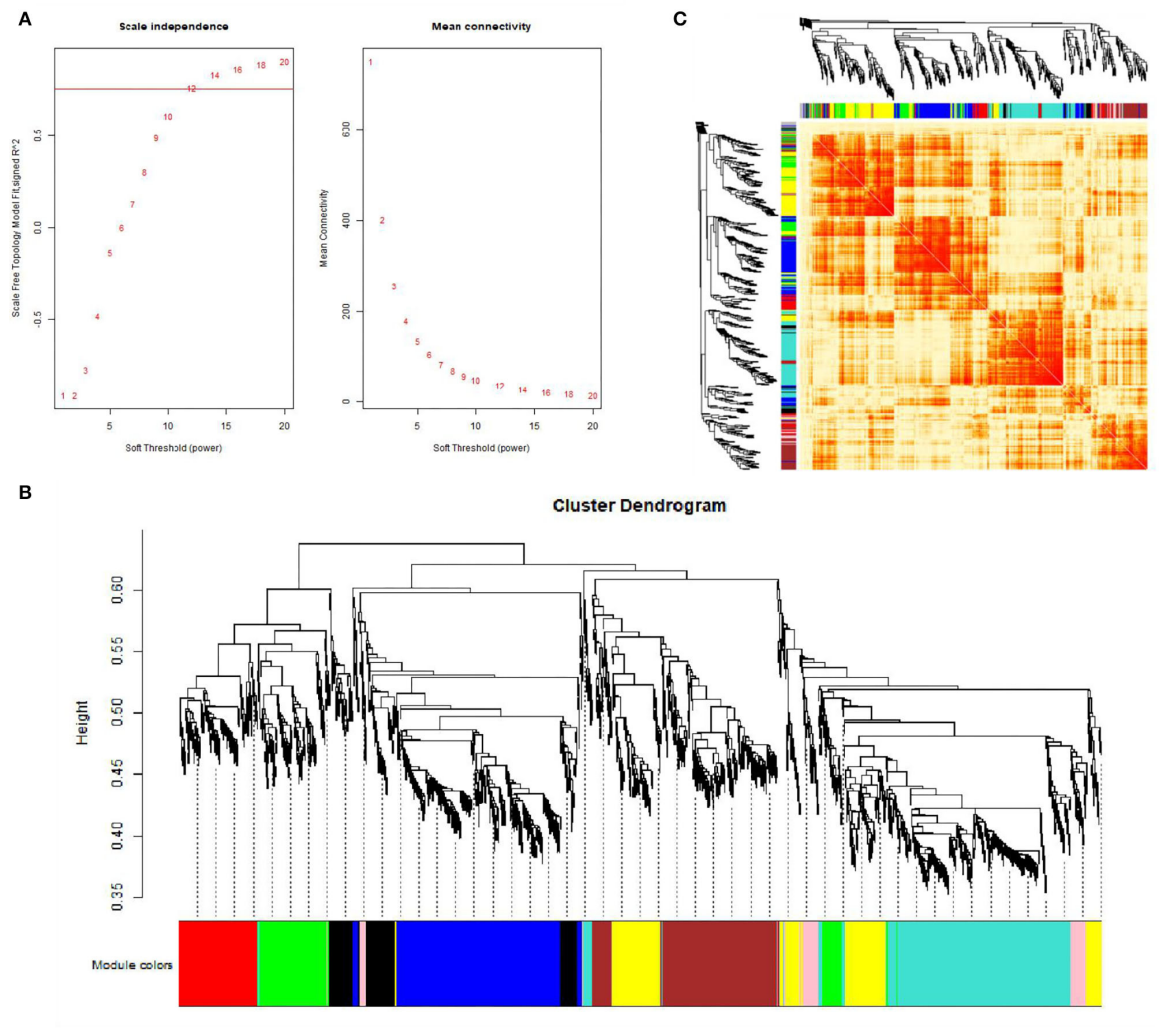
Gene co-expression networks in subcutaneous adipose tissue (GSE116775). **(A)** Analysis of the scale-free fit index for soft-thresholding powers (left) and the mean connectivity for various soft-thresholding powers (right); **(B)** Gene expression clustering tree and recognition module in co-expression network; **(C)** Network heatmap plot in the co-expression modules.

This study performed a WGCNA analysis based on the GSE116775 dataset. The clustering analysis of the selected samples is shown in Figure 1. A total of 185 samples were divided into four independent clusters, each corresponding to a tissue type, indicating that different tissue types were the main reason for the differences between samples, and thus the results of the analysis are expected to uncover modules and Hub genes specific to bovine subcutaneous adipose tissue. When 0.8 is used as the correlation coefficient threshold, the soft threshold power is selected as 12 (Figure 2A). Then, on the basis of the determined soft threshold, the weighted gene co-expression network is constructed, the co-expression modules are divided by dynamic cutting and module merging, and finally, nine co-expression modules are obtained. The modules with the most genes were turquoise modules (1,509), followed by blue modules (1,387), brown modules (1,157), and yellow modules (1,058) (Figure 2B). In addition, we have drawn adjacency heat maps of all the analyzed genes, which show that these modules are independent of other modules (Figure 2C).

### Identification of Key Modules

**Figure 3:**
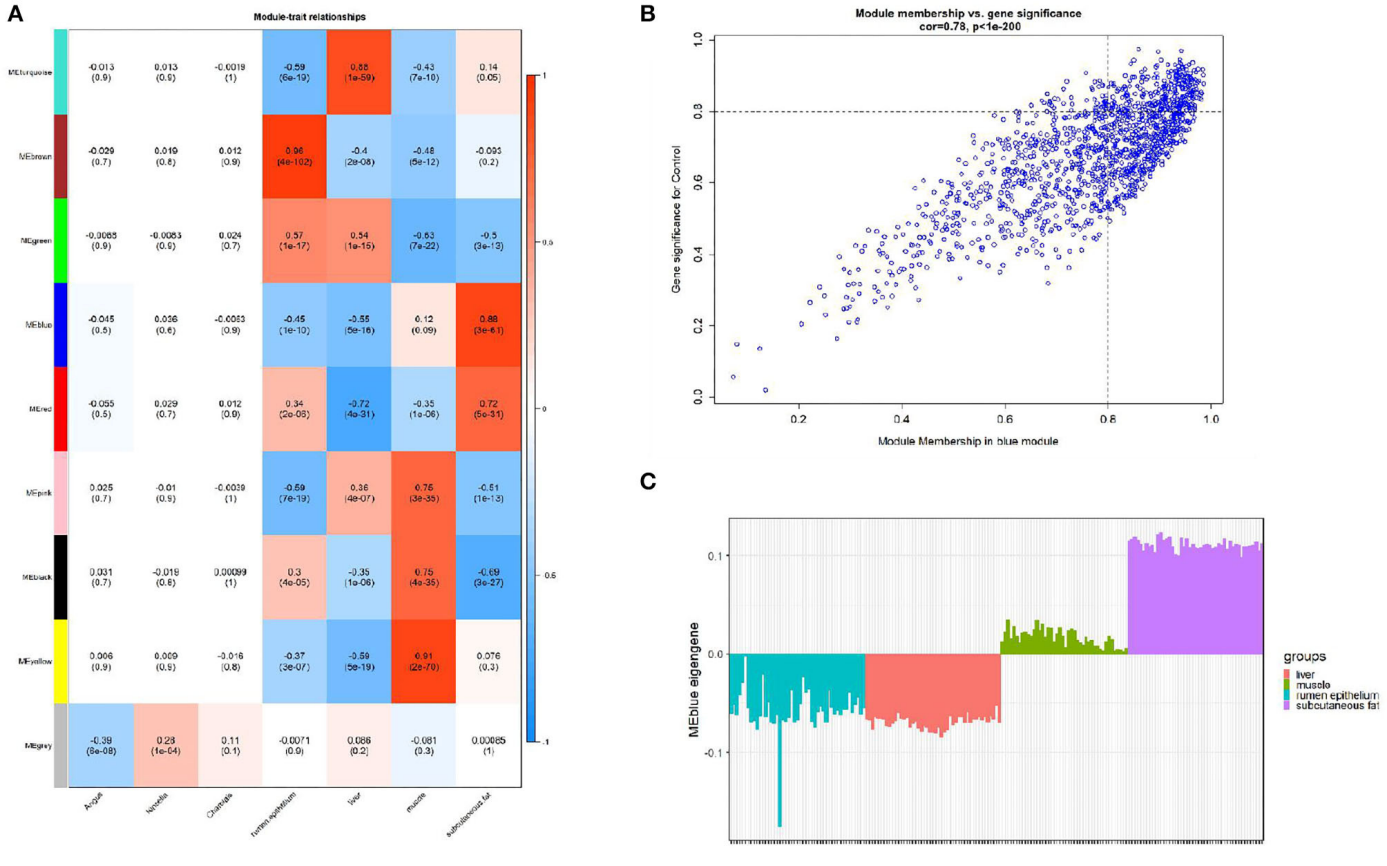
Module-trait correlations analysis in subcutaneous adipose tissue (GSE116775). **(A)** The correlation heat map between the module and subcutaneous adipose tissue (each unit contains the correlation coefficient and the corresponding P value); **(B)** The significance of genes related to subcutaneous adipose tissue in the blue module (a dot represents the genes in the blue module); **(C)** Module eigengene (y-axis) across samples (x-axis) from the blue module (associated to Subcutaneous fat tissue).

We calculated the correlation coefficient and the corresponding statistical significance between modular feature genes and clinical traits and displayed the results with a heat map (Figure 3A). According to the aim of the study, we found the highest correlation between the blue module and bovine subcutaneous adipose tissue (*r* = 0.88, *p* = 3e-61). Figure 3B shows that the genes in the blue module are of great significance for the study of subcutaneous fat (Supplementary Table 4). Figure 3C shows that the expression level of genes related to subcutaneous adipose tissue in the blue module is significantly higher than that in other tissues, so the blue module is selected for further analysis.

### Functional Annotation of the Key Co-Expression Module

**Figure 4:**
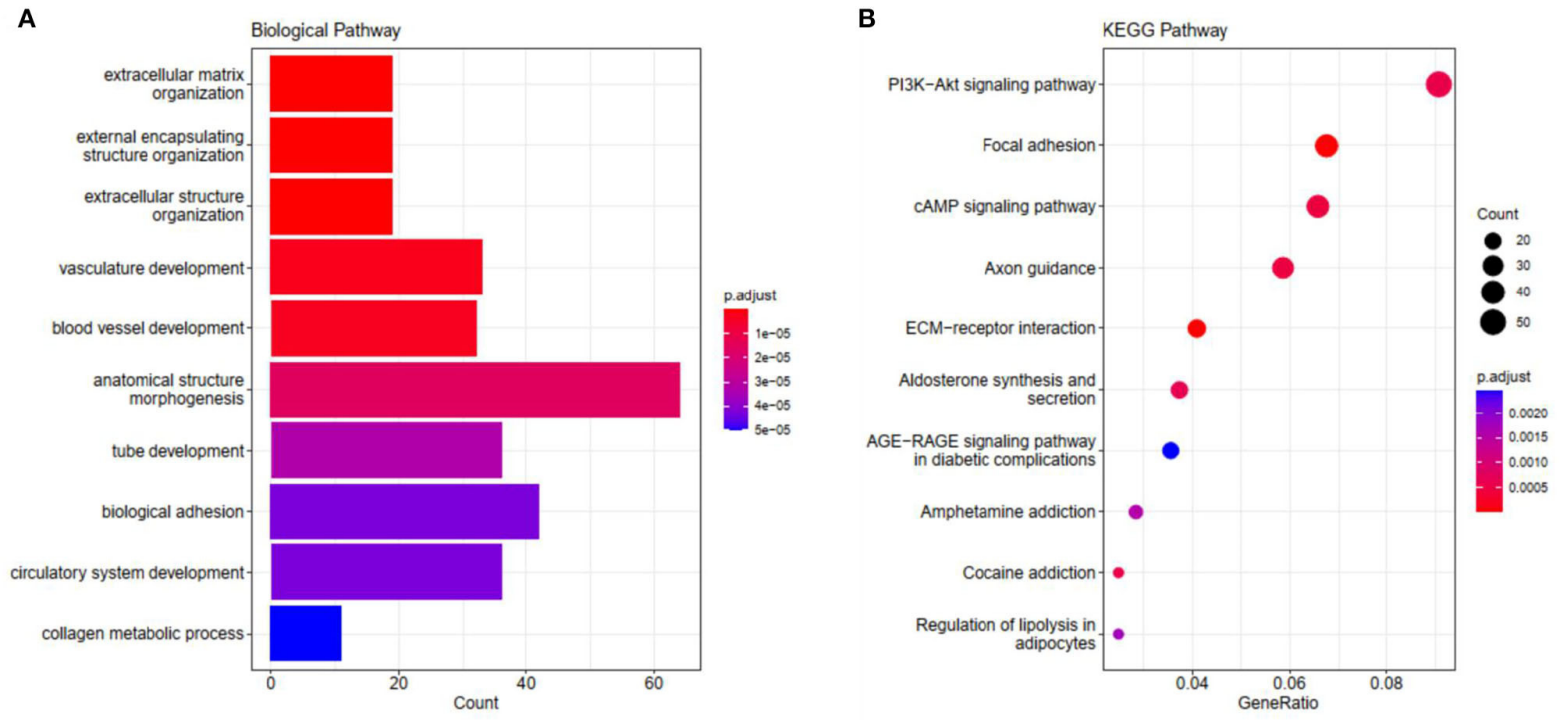
The visualization results of **(A)** partial GO biological function analysis and **(B)** partial KEGG analysis of the blue module. The top 10 significant enriched pathways were shown.

We carried out GO and KEGG pathway enrichment analysis of the genes in the blue module in order to find out the main biological processes and signal pathways of enrichment. Figure 4 shows the first 10 terms for GO-BP and KEGG enrichment analysis (all enriched terms and explanations for the first 10 terms can be found in Supplementary Tables 5, 6). GO-BP functional enrichment analysis showed that the genes in the blue module were mainly enriched in extracellular matrix tissue, bioadhesion, and collagen metabolism (Figure 4A). KEGG analysis showed that ECM receptor interaction, focal adhesions, cAMP signal pathway, PI3K-AKT signal pathway and the regulation of lipolysis of adipocytes were the obvious ways of gene enrichment in the blue module (Figure 4B).

### Excavation of the Hub Gene

**Figure 5:**
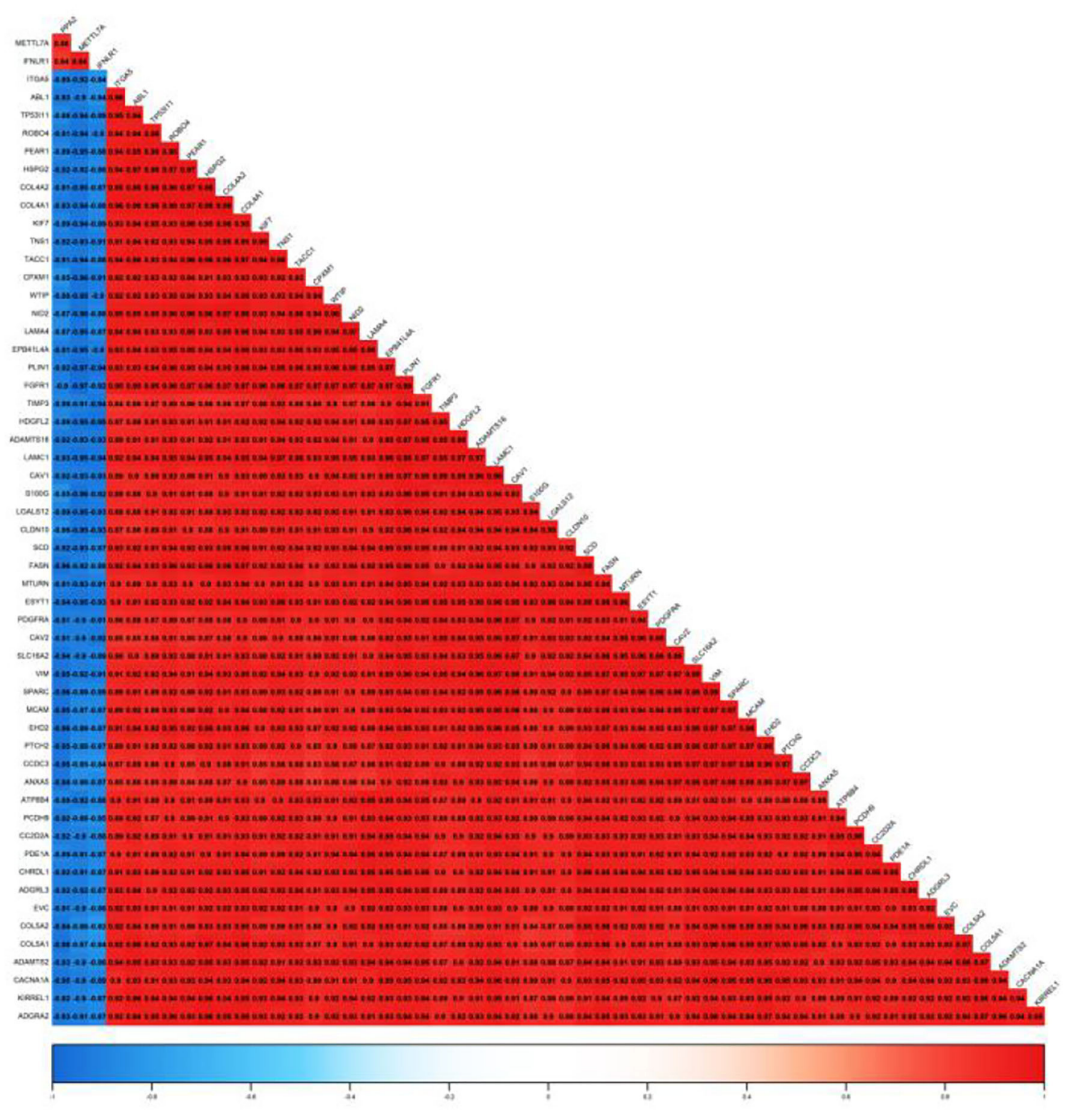
Correlation of the top 56 genes with high MM and GS in the blue module.

**Figure 6:**
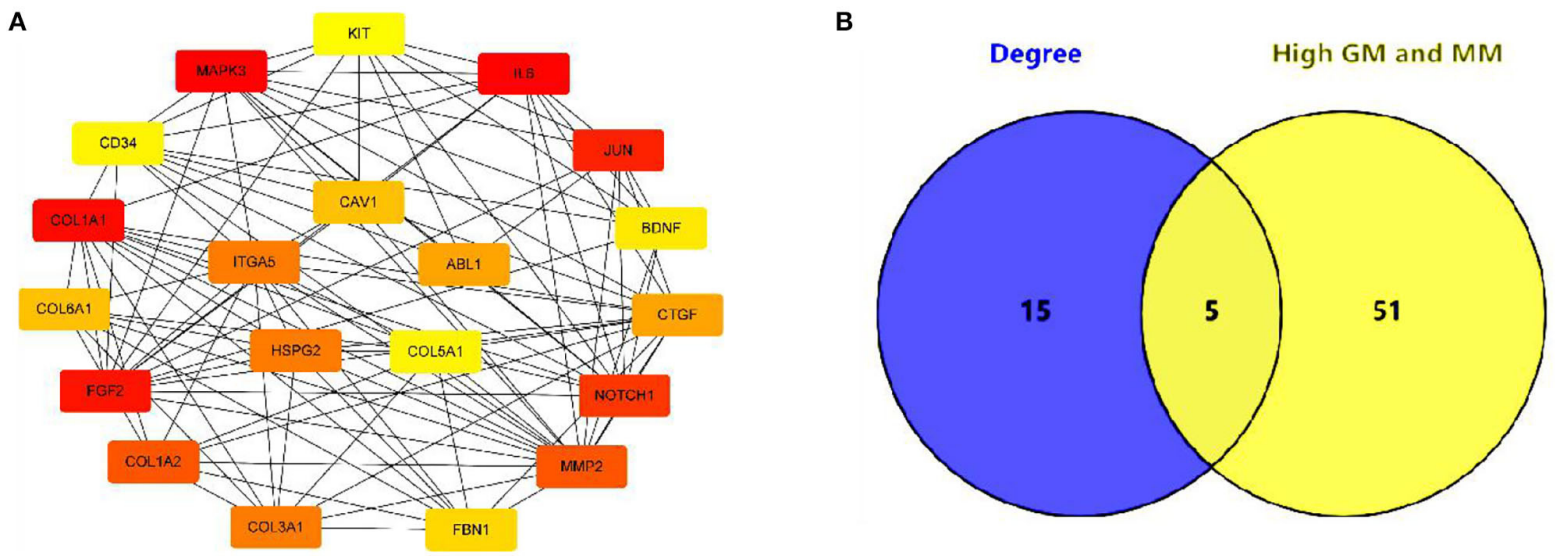
Identification of Hub gene. **(A)** The top 20 genes with the highest connectivity identified by Cytoscape software; **(B)** Identify the common genes in the co-expression network and PPI network.

In this study, 56 genes with high clinical trait relationships (GS ≥ 0.88) and high linkage (MM ≥ 0.92) in the blue module were selected as pivotal genes for WGCNA (Supplementary Table 7; Figure 5). According to the STRING database, all the genes in the module are used to build the PPI network using Cytoscape software (Supplementary Table 8; Figure 6A), and the 20 genes with the highest connectivity are defined as the Hub genes in the PPI network. Then, the five common genes (CAV1, ITGA5, COL5A1, ABL1, and HSPG2) in the co-expression network and PPI network are defined as “real” Hub genes (Figure 6B).

### Induction of Differentiation of Bovine Subcutaneous Adipocytes

**Figure 7:**
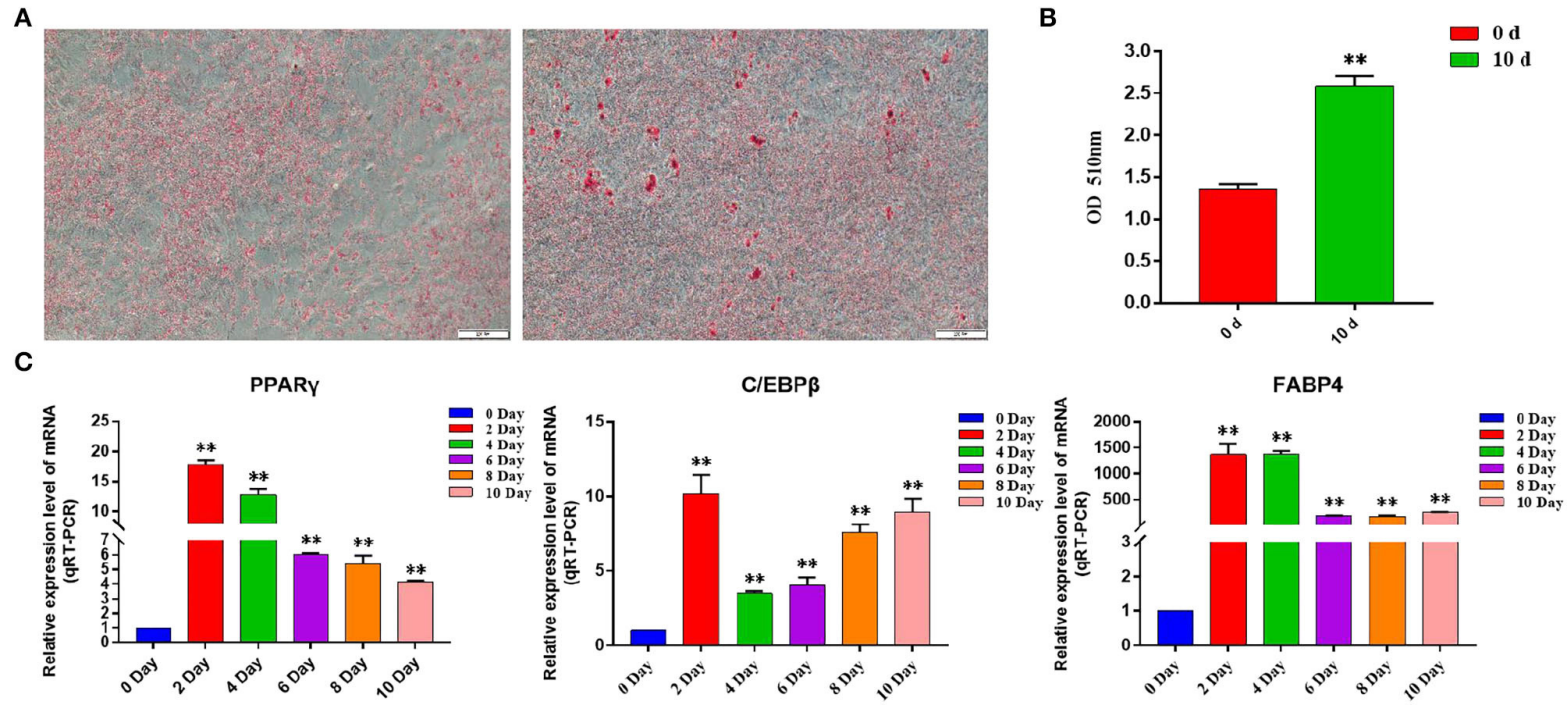
Induction of primary adipocyte differentiation. **(A)** Bovine adipocytes were stained with oil red O at 0 d (left) and 10 d (right) after adipogenic differentiation; **(B)** Absorbance measurements at 510 nm. Control: isopropyl alcohol, 0 d: adipocyte extracts from 0 day of induced differentiation, 10 d: adipocyte extracts from 10 day of induced differentiation; **(C)** Expression of adipogenic marker genes in the process of adipogenic differentiation. Compared with the control, “*” means significant difference (*p* < 0.05), and “**” means extremely significant difference (*p* < 0.01).

By inducing the differentiation of bovine subcutaneous adipocytes, qRT-PCR and oil red O staining were used to detect the sequential expression of adipogenic marker genes in the induced cells for 0–10 days. The results showed that compared with that before induction, the expression levels of PPARγ, C/EBPβ, and FABP4 increased significantly after induction (*p* < 0.01) (Figure 7C), and the content of lipid droplets in cells increased significantly (*p* < 0.01) (Figures 7A,B), indicating that the model of inducing differentiation of bovine subcutaneous adipocytes was successfully established.

### Validation of the Hub Genes

**Figure 8:**
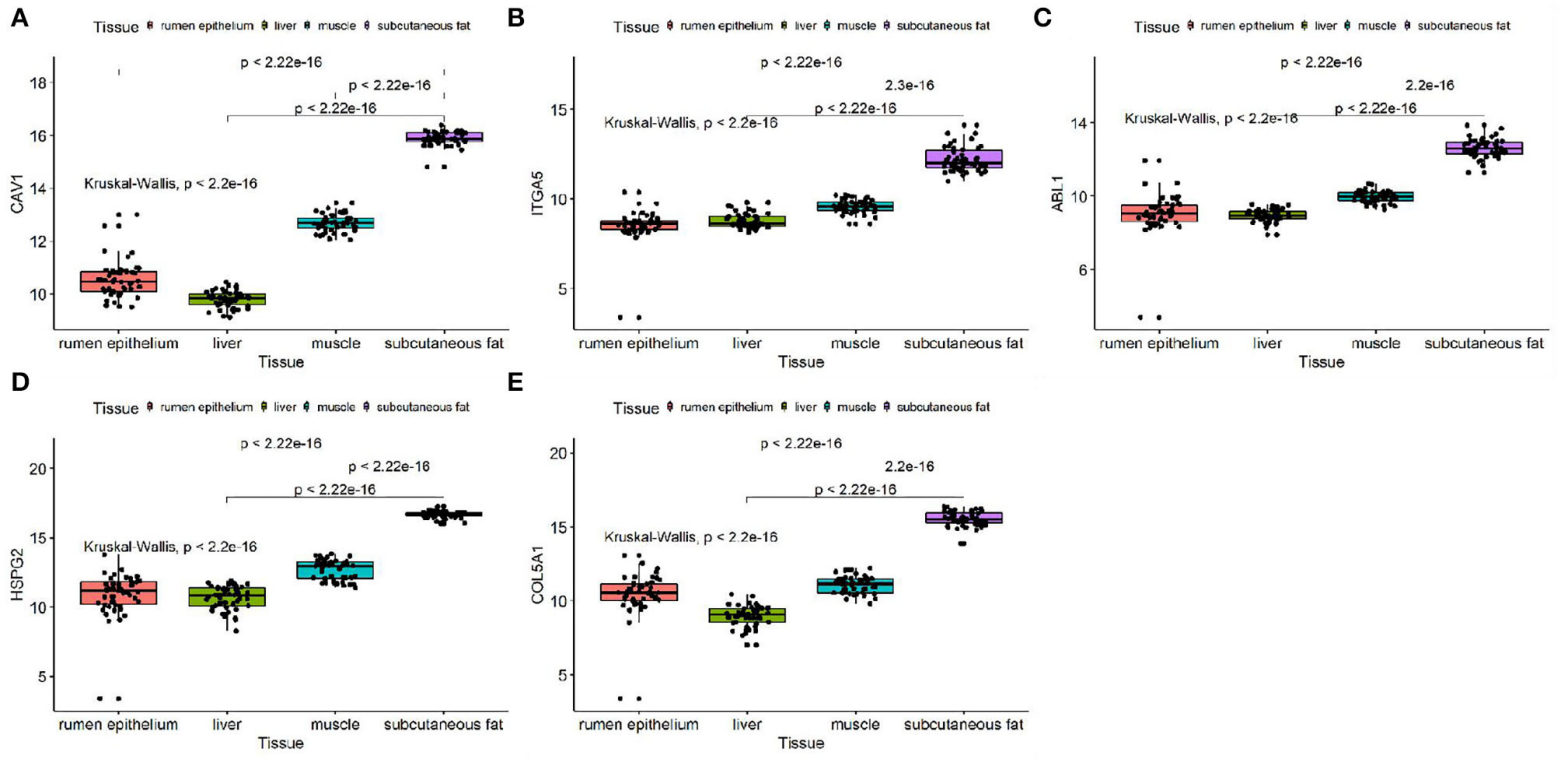
Expression of Hub genes in dataset GSE116775. **(A–E)** Expression levels of **(A)** CAV1, **(B)** ITGA5, **(C)** ABL1, **(D)** HSPG2, and **(E)** COL5A1 were significantly increased in Subcutaneous fat tissue.

**Figure 9:**
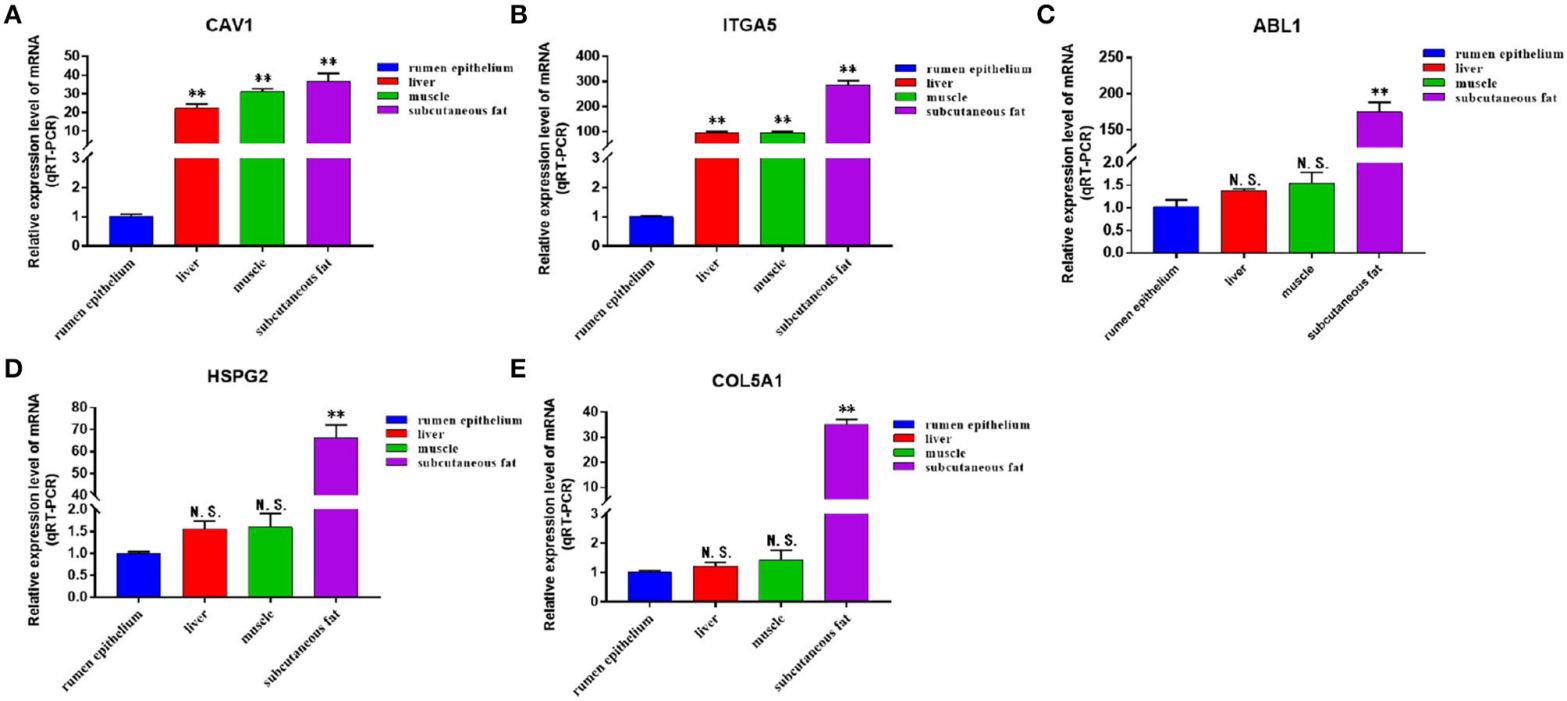
The expression level of Hub gene in different tissue samples. **(A–E)** Compared with other tissues, the expression levels of **(A)** CAV1, **(B)** ITGA5, **(C)** ABL1, **(D)** HSPG2, and **(E)** COL5A1 in subcutaneous adipose tissue were significantly increased. Compared with the control, “*” means significant difference (*p* < 0.05), “**” means extremely significant difference (*p* < 0.01), and “N.S.” indicates non-significant difference.

**Figure 10:**
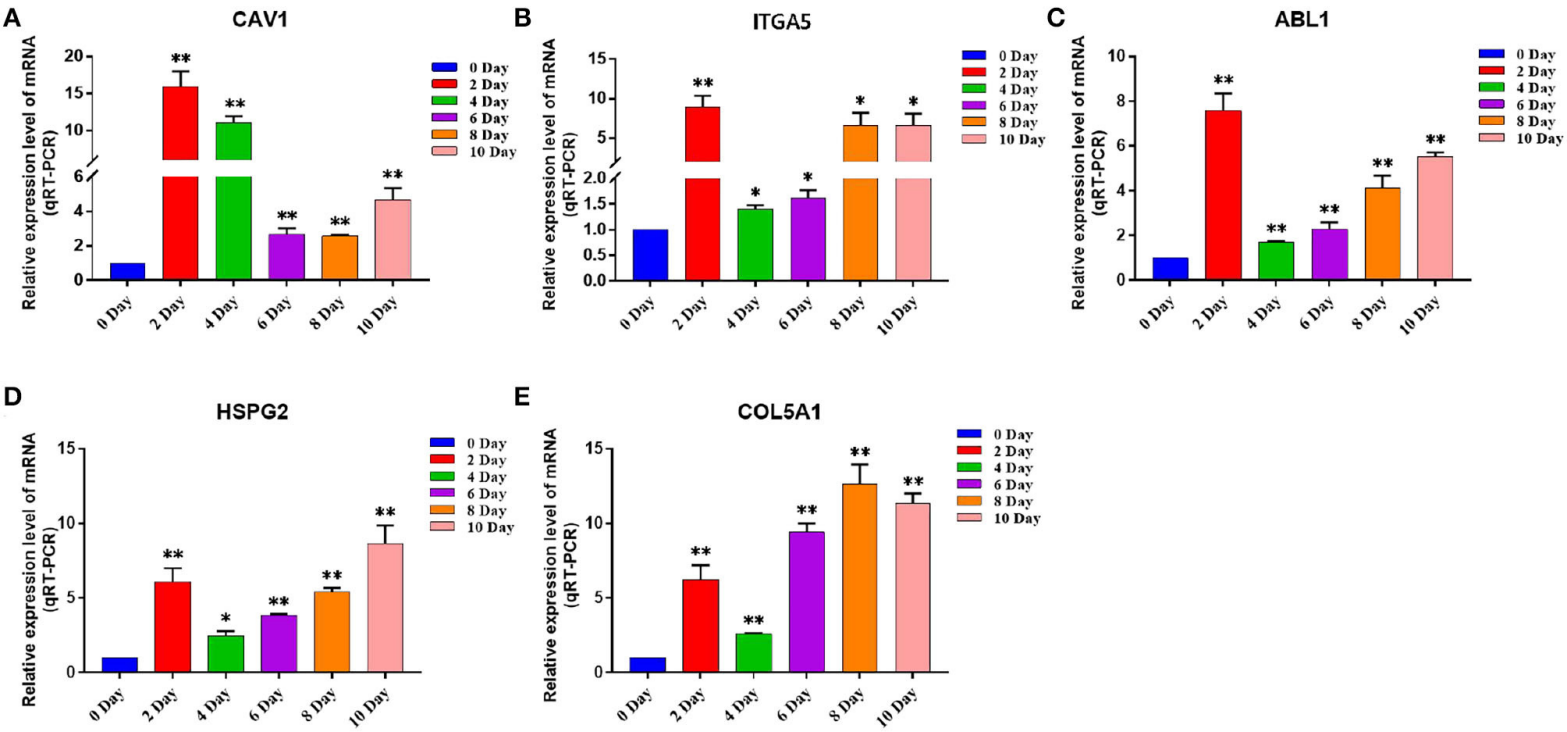
The expression level of Hub gene in different differentiation stages of adipocytes. **(A–E)** Compared with that before induction, the expression levels of **(A)** CAV1, **(B)** ITGA5, **(C)** ABL1, **(D)** HSPG2, and **(E)** COL5A1 were significantly increased after induction. Compared with the control, “*” means significant difference (*p* < 0.05), “**” means extremely significant difference (*p* < 0.01), and “N.S.” indicates non-significant difference.

We discussed the differential expression level of Hub gene in the data set. The results showed that Hub gene expression was significantly higher in subcutaneous adipose tissue than in rumen epithelium, liver, and muscle tissue (*p* < 0.01) (Figure 8). QRT-PCR was used to detect the expression level of the Hub gene in tissues and induced subcutaneous adipocytes. The results showed that the Hub gene was highly expressed in adipose tissue (Figure 9); and compared with that before induction, the expression level of the Hub gene increased significantly after cell induction (*p* < 0.01) (Figure 10). These results suggest that these Hub genes play an important role in the study of adipose tissue development.

## Discussion

Fat deposition is a qualitative trait typically regulated by multiple genes that function by interacting with each other. In traditional unidimensional studies, it is difficult to find the key genes for this trait and their mechanisms of action. However, WGCNA is a powerful statistical method based on gene correlation that can be used to construct gene networks, detect modules, identify pivotal genes, and screen candidate genes as biomarkers (10). In the statistical process, WGCNA focuses on dealing with a group of gene modules rather than individual genes, which avoids the disadvantage of only dealing with single genes and neglecting molecular transcriptional networks. Therefore, using WGCNA to study the co-expression network of multiple tissue phenotypes can identify the marker genes in subcutaneous adipose tissue and provide a research basis for exploring the molecular mechanism of fat deposition.

**Figure 11:**
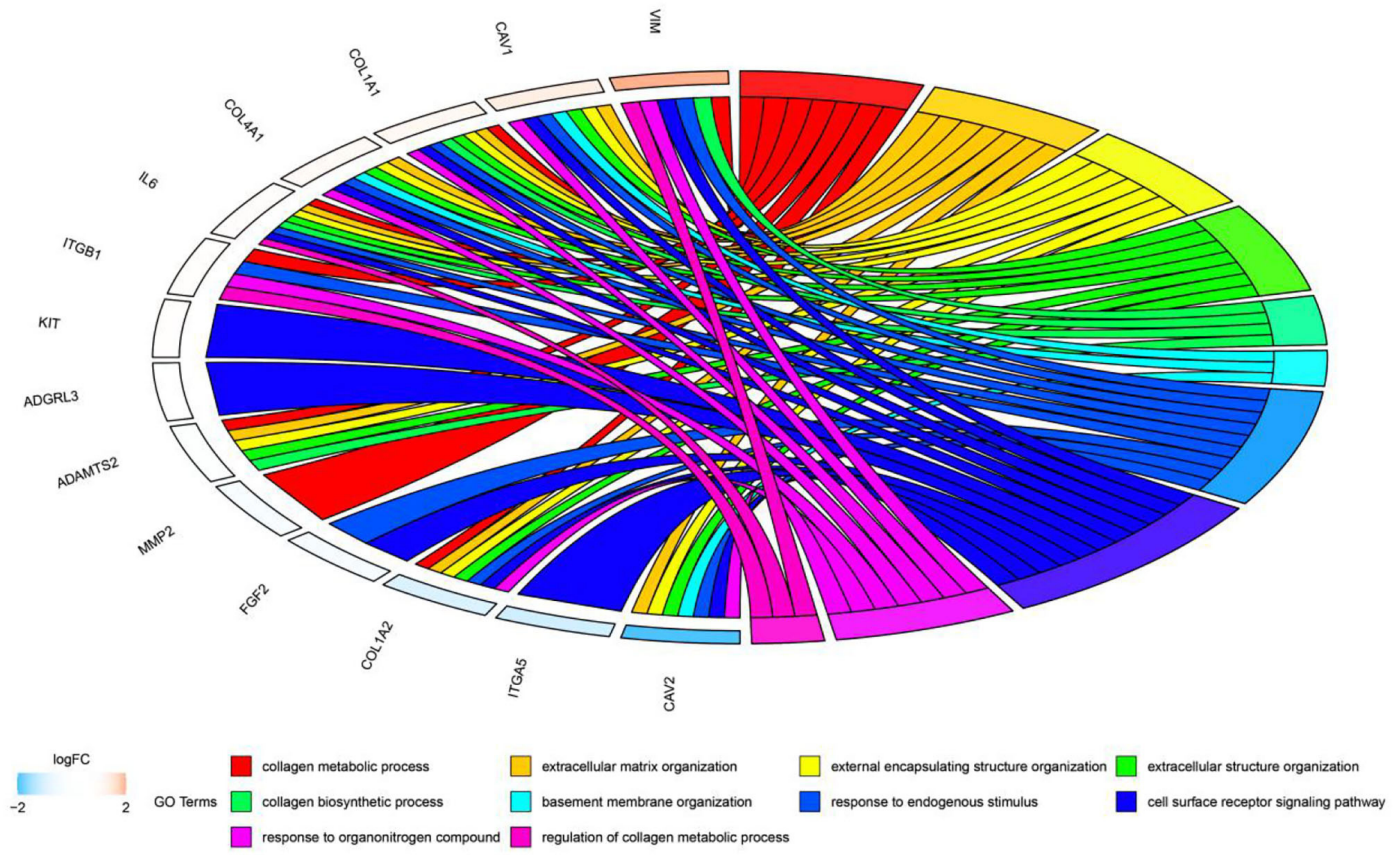
Circos plot to indicate the relationship between hub genes and KEGG pathways.

In this study, we downloaded 185 samples belonging to the GSE116775 dataset from the GEO database, and obtained nine modules by using the WGCNA method. According to the correlation study of the topological overlap matrix (TOM) graph (Figure 2), each module is proved to be independent of the other modules. In addition, after trait-module correlation analysis, the blue module was identified as the key module positively related to subcutaneous adipose tissue, which means that the gene expression in this module is closely related to subcutaneous adipose tissue. Subsequent GO analysis showed that the genes in fat-related modules were mainly enriched in the extracellular matrix organization, biological adhesion, collagen metabolic process, and other biological functions. KEGG enrichment showed that these genes were mainly involved in ECM receptor interaction, focal adhesions, cAMP signal pathway, PI3K-AKT signal pathway, and the regulation of lipolysis of adipocytes. In addition, adipose tissue-related “real” Hub genes (CAV1 and ITGA5) are mainly involved in KEGG pathways related to extracellular matrix organization and cell surface receptor signaling transduction pathways (Figure 11). Through literature research, we found that the composition and distribution of the extracellular matrix changed in the process of adipocyte differentiation, suggesting that organizing the components of the extracellular matrix into a suitable structure is a necessary condition for adipocyte differentiation and maintenance. Among the extracellular matrix components, the extracellular network of fibronectin (FN) is the first to form but gradually degrades as adipocytes differentiate. The type I collagen network is the last to form and remains well organized during the later stages of adipocyte differentiation. The extracellular network of type III, V, and VI collagen was formed in the middle stage of adipocyte differentiation and maintained until the late stage of adipocyte differentiation. The reticular structures of type IV collagen and laminin were degraded during differentiation and located on the surface of globular cells (18). And with regard to adipose tissue regeneration, appropriate ECM may be needed to support cell attachment, proliferation, and differentiation until adipocytes can secrete their own ECM (19). In addition, ECM is also an important part of the focal adhesions pathway and PI3K-AKT pathway. Focal adhesions are cell-matrix adhesion structures mediated by integrins whose functions include anchoring the ends of actin filaments, promoting strong attachment to the matrix, and functioning as an integrin signaling platform (20). A PI3K-AKT signaling pathway is involved in many biological processes, including cell cycle, apoptosis, angiogenesis, and glucose metabolism, which is essential for cell proliferation and apoptosis (21, 22). And the cAMP signaling pathway has been reported to play an important role in the regulation of energy homeostasis with its involvement in adipogenesis and lipid metabolic processes (23–26).

With regard to the identified central genes, the functional studies of CAV1, ITGA5, COL5A1, ABL1, and HSPG2 in the process of fat deposition are insufficient. Caveolae are small flask-shaped invaginations of the plasma membrane that are found with remarkable abundance in endothelial cells, myotubes, and adipocytes (27). They are considered a subset of the so-called lipid raft domains and segregate a number of membrane-related processes (28). The Caveolae protein family has three highly related members (caveolin-1 through-3). In adipocytes, Caveolin-1 expression was found to be a key step in increasing caveolae density, increasing the ability of adipocytes to accommodate larger lipid droplets, and promoting cell expansion through increased glucose utilization (29). COL5A1 and ITGA5 are important constituents of ECM. COL5A1 is differentially expressed before and after bariatric surgery, which may be a new candidate gene for regulating adipose tissue function (30). ITGA5 is expressed in different degrees in mesenchymal stem cells isolated from adipose tissue and white mature adipocytes, which is related to the Hippo pathway, while the Hippo pathway controls tissue growth and regulates cell proliferation, differentiation, and cell death (31–33). ABL1 is highly expressed in the subcutaneous fat of obese humans and high-fat diet-induced obese mice, and it is able to regulate diet-induced obesity by improving insulin sensitivity in subcutaneous fat (34). HSPG2 is a heparan sulfate proteoglycan, which is a component of the basement membrane and participates in a variety of biological activities. It was found that the mass and cell size of white adipose tissue in HSPG2 knockout mice were smaller than those in the control group, which may regulate the catabolism of lipids and glucose by transforming the composition of muscle fibers into oxidized fibers (35).

Adipose tissue is mainly composed of adipocytes and matrix components around the cells. Peroxisome proliferator-activated receptor gamma (PPARγ) and CCAAT enhanced binding protein (C/EBP) have been proved to be the main regulatory factors involved in adipocyte differentiation. Adipocytes rapidly induce the expression of C/EBPβ and C/EBPδ proteins within 4 h of induced differentiation and then activate the transcription of PPARγ and CEBPα (36). The expression of PPARγ and C/EBPα regulates a variety of genes in coordination, which determines the phenotype of cell differentiation (37). Fatty acid binding protein 4 (FABP4) gene is highly expressed during adipocyte differentiation and can bind and transport long-chain fatty acids, which plays an important role in the synthesis and decomposition of triglycerides. In this study, by constructing the differentiation model of bovine subcutaneous adipocytes, the expression level of the adipogenic marker gene and the formation of lipid droplets were detected by the qRT-PCR technique. The results showed that compared with that before induction, the expression levels of adipogenic marker genes PPARγ, C/EBPα and FABP4 in induced subcutaneous adipocytes were all at a high level, and the number of lipid droplets increased significantly after induction, which proved that we successfully established the induced differentiation model of bovine subcutaneous adipocytes. Subsequently, the expression of Hub gene (CAV1, ITGA5, COL5A1, ABL1, and HSPG2) in bovine subcutaneous adipocytes was detected, and it was found that the expression level of five Hub genes increased significantly after cell induction, especially on the second day of induction, which was consistent with the expression of adipogenic marker genes, which may be due to the effect of changing induction medium to stimulate adipocyte differentiation. It is suggested that these five Hub genes may play the same role as adipogenic marker genes in the process of adipocyte differentiation.

In summary, CAV1, ITGA5, COL5A1, ABL1, and HSPG2 from the blue module are candidate genes related to subcutaneous adipose tissue, and their expression levels are related to adipocyte proliferation and differentiation, which may regulate adipose tissue growth and development by mediating extracellular matrix tissue and cell surface receptor signal transduction pathway. Although many studies have reported that these genes play an important role in fat development, the specific mechanism is not clear. Finally, we verified our results using tissue samples and adipocyte samples obtained by real-time fluorescence quantitative PCR and found that these Hub genes were highly expressed in adipose tissue and induced differentiated adipocytes. Therefore, these five candidate genes can be regarded as new biomarkers of subcutaneous adipose tissue. The results of this study will provide a new perspective on adipose tissue development and adipose deposition.

## Data Availability Statement

The original contributions presented in the study are included in the article/Supplementary Material, further inquiries can be directed to the corresponding author.

## Ethics Statement

The animal study was reviewed and approved by the Animal Welfare Committee of Ningxia University.

## Author Contributions

HS and CP: made the same contribution to the work and analyzed the data. YM and HS: conceived and designed the research. HS: wrote the manuscript. SW, CY, JZ, CH, HH, XF, MY, ZL, YG, ZW, and YM: modified the manuscript. All authors read and approved the final manuscript.

## Funding

This study was supported by grants from the National Natural Science Foundation of China (32072720-31672403), the Leading Talents Fund in Science and Technology Innovation in Henan Province (No. 194200510022), the Key Research and Talent Introduction Project of Ningxia Hui Autonomous Region (2019YCZX0068, 2021BEF01002, and 2021NXZD1), and the Cultivation Project for Talents in Science and Technology Innovation of Ningxia Hui Autonomous Region (2020GKLRLX02). The funding bodies played no role in the design of the study, collection, analysis, and interpretation of data and writing the manuscript.

## Conflict of Interest

The authors declare that the research was conducted in the absence of any commercial or financial relationships that could be construed as a potential conflict of interest.

## Publisher's Note

All claims expressed in this article are solely those of the authors and do not necessarily represent those of their affiliated organizations, or those of the publisher, the editors and the reviewers. Any product that may be evaluated in this article, or claim that may be made by its manufacturer, is not guaranteed or endorsed by the publisher.
