# Supplementary material for: Weighted Gene Co-Expression Network Analysis Identifies Key Modules and Central Genes Associated With Bovine Subcutaneous Adipose Tissue
Source: Front Vet Sci. 2022 Jun 22;9:914848. doi: 10.3389/fvets.2022.914848 (PMC9257221; doi:10.3389/fvets.2022.914848)
Supplement: Supplementary file 1 [file Data_Sheet_1.ZIP › supplementary table/supplementary table 2íó3.docx]

**Supplementary Table 2： Nucleotide sequences of the primers used for Real-time fluorescence quantitative PCR**

| Genes | Direction | Primer sequence (5’-3’) | Amplification size |
| --- | --- | --- | --- |
| CAV1 | F | CCTTAAATCACAGCCCAGGGAA | 84bp |
|  | R | TGAGTCTACGTATTTGCCCCC |  |
| ITGA5 | F | CGTTTGCTGTCTGCCCTCTTTG | 140bp |
|  | R | ACACGGCTGATGCACTGAATCT |  |
| COL5A1 | F | TGGCTACGGCGAAGGCATTG | 113bp |
|  | R | GGTTGGAGTCGTTGGAGGTGAG |  |
| ABL1 | F | GGGGAATGTGAAATCCCACG | 115bp |
|  | R | CTGCTGCCCCATAAAGCAAG |  |
| HSPG2 | F | CAGATGCCCCATCGGCTATT | 143bp |
|  | R | GGCCATACACAGGGTCACAA |  |
| Β-actin | F | TCGGTTGGATCGAGCATTCC | 141bp |
|  | R | GTGGCTTTTGGGAAGGCAAA |  |

**Supplementary Table 3： Real-time fluorescence quantitative reaction program**

Table 3-3 qRT-PCR reaction program

| Procedure | Temperature | Time | Cycles |
| --- | --- | --- | --- |
| Preincubation | 95℃ | 600 s | 1 |
|  | 95℃ | 10 s |  |
| 3 Step Amplification | 63℃ | 20 s | 35 |
|  | 72℃ | 15 s |  |
|  | 95℃ | 10 s |  |
| Melting | 65℃ | 60 s | 1 |
|  | 97℃ | 1 s |  |
| Cooling | 97℃ | 30 s | 1 |
