# Supplementary figures and images for: Weighted Gene Co-Expression Network Analysis Identifies Key Modules and Central Genes Associated With Bovine Subcutaneous Adipose Tissue
Source: Front Vet Sci. 2022 Jun 22;9:914848. doi: 10.3389/fvets.2022.914848 (PMC9257221; doi:10.3389/fvets.2022.914848)

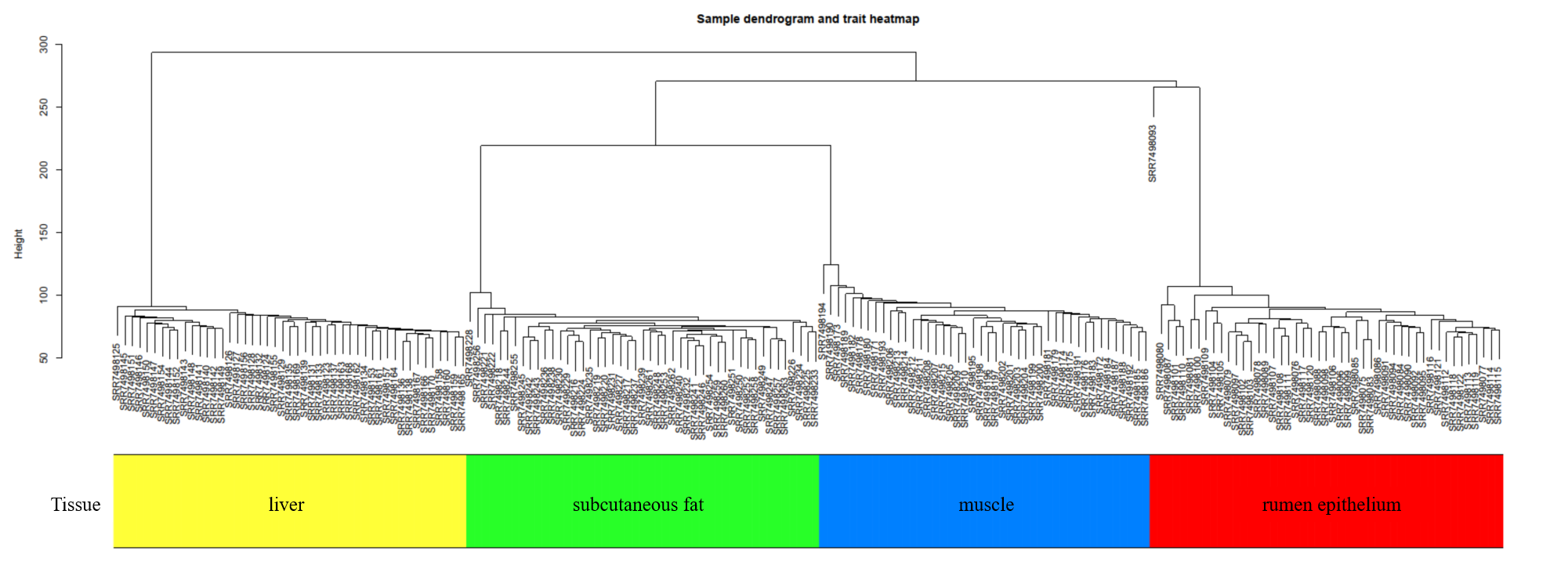

Supplement: Supplementary file 2 [file Data_Sheet_2.ZIP › 914848-Raw Data/figure/figure-1.png]

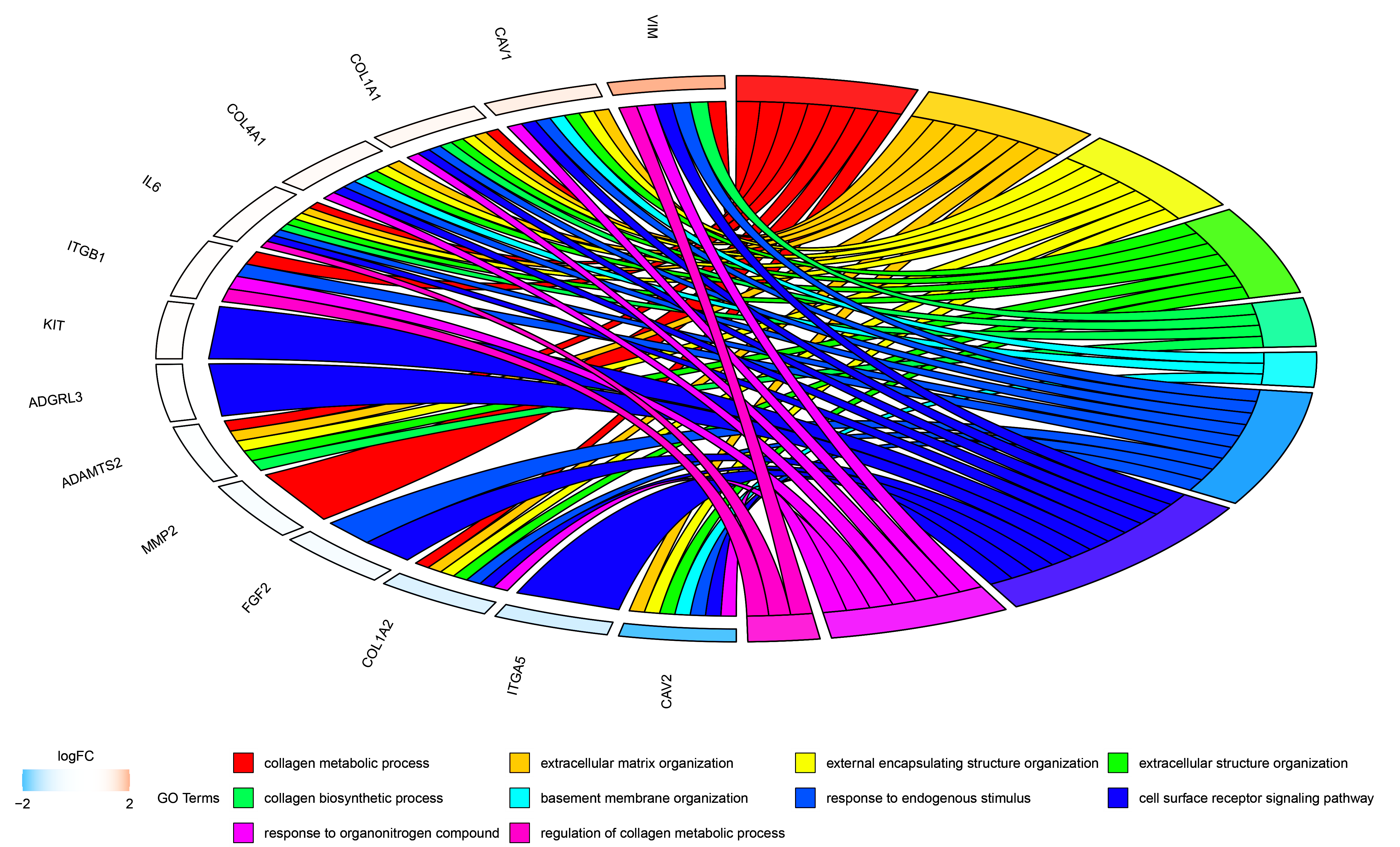

Supplement: Supplementary file 2 [file Data_Sheet_2.ZIP › 914848-Raw Data/figure/figure-11.png]

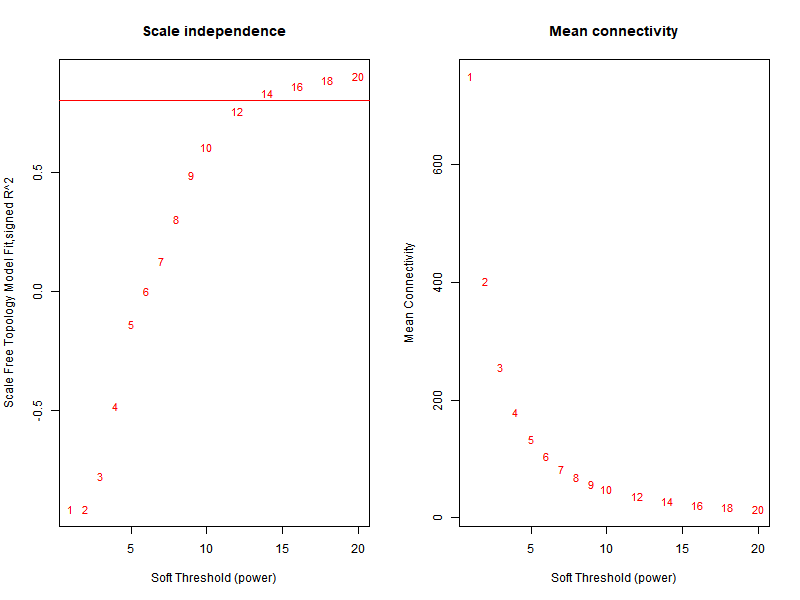

Supplement: Supplementary file 2 [file Data_Sheet_2.ZIP › 914848-Raw Data/figure/figure-2A.png]

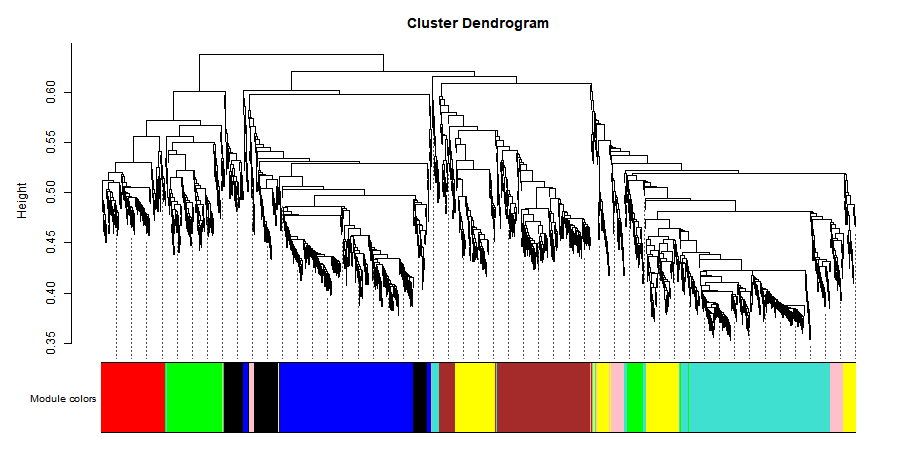

Supplement: Supplementary file 2 [file Data_Sheet_2.ZIP › 914848-Raw Data/figure/figure-2B.png]

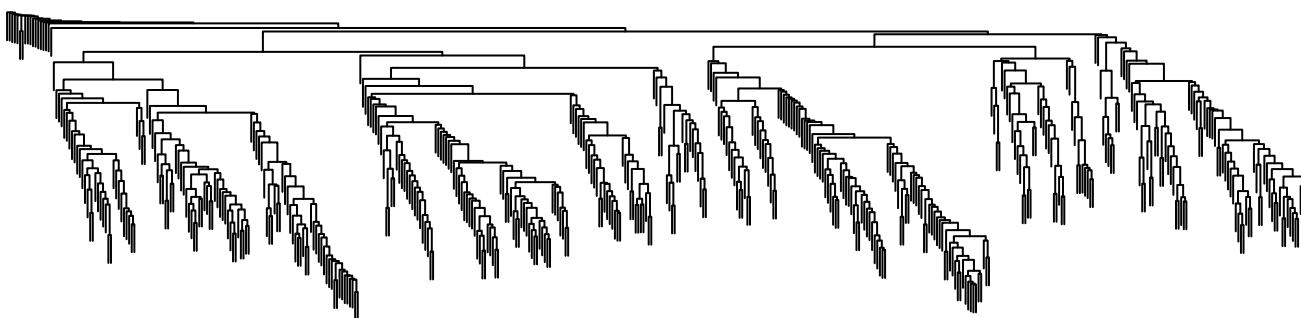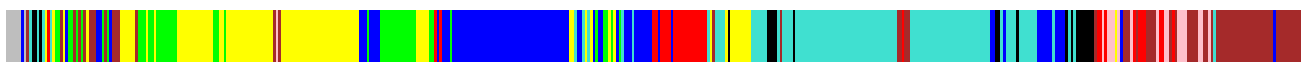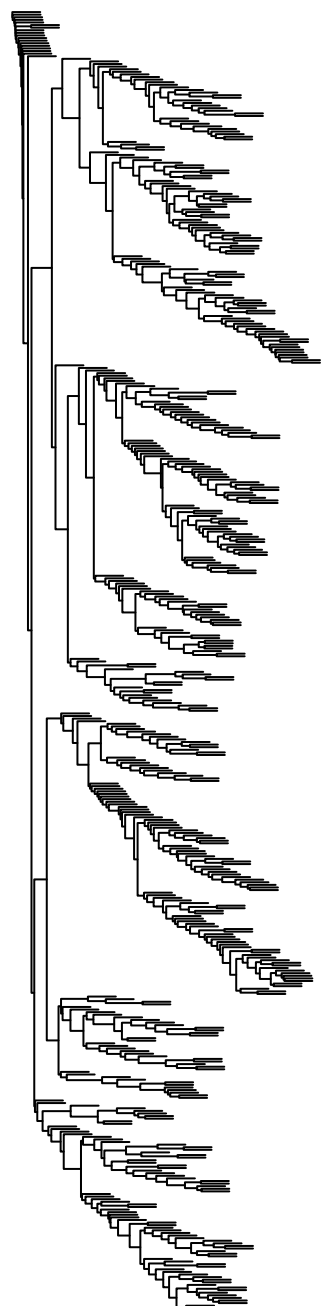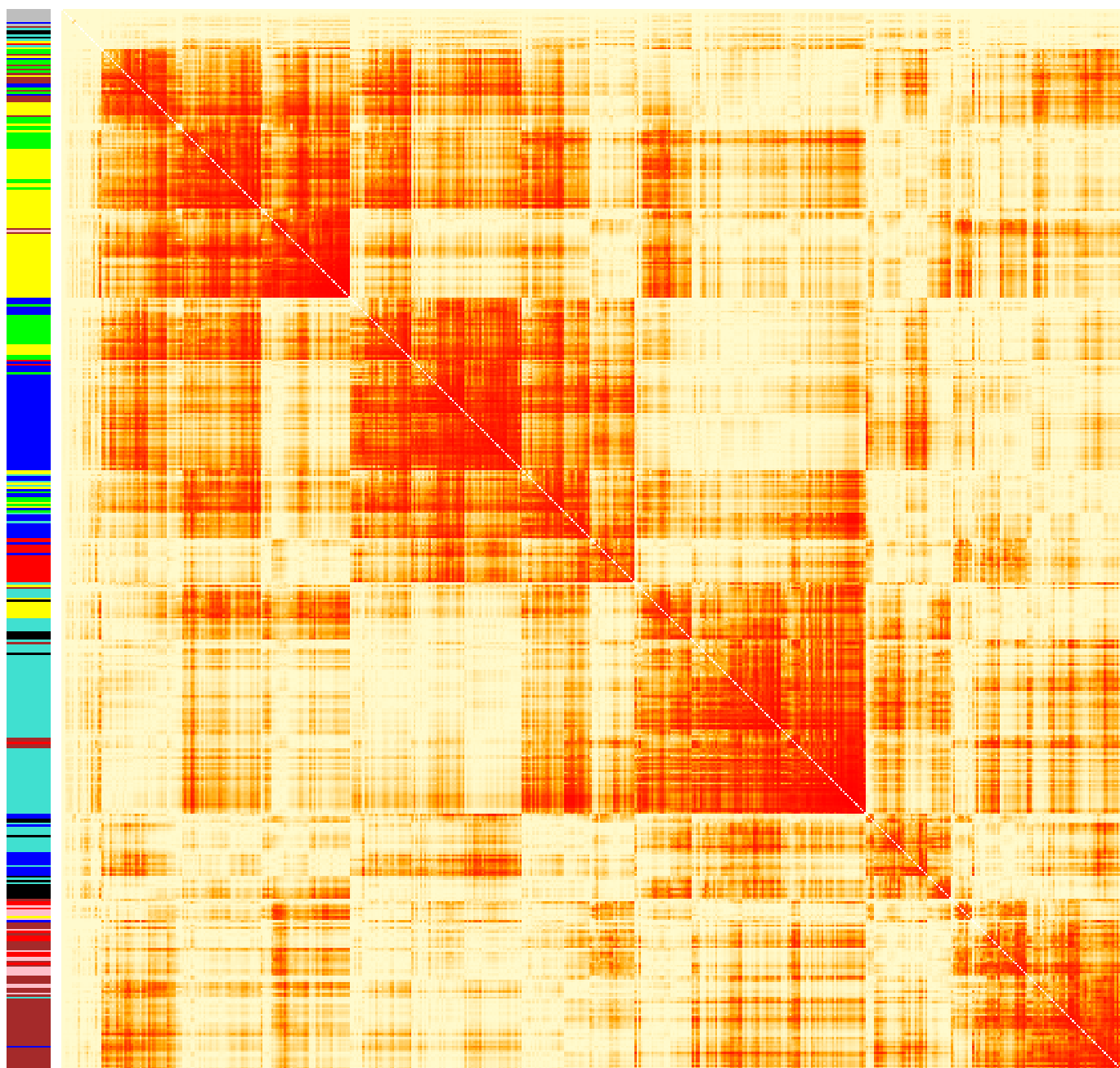

Supplement: Supplementary file 2 [file Data_Sheet_2.ZIP › 914848-Raw Data/figure/figure-2C.pdf]

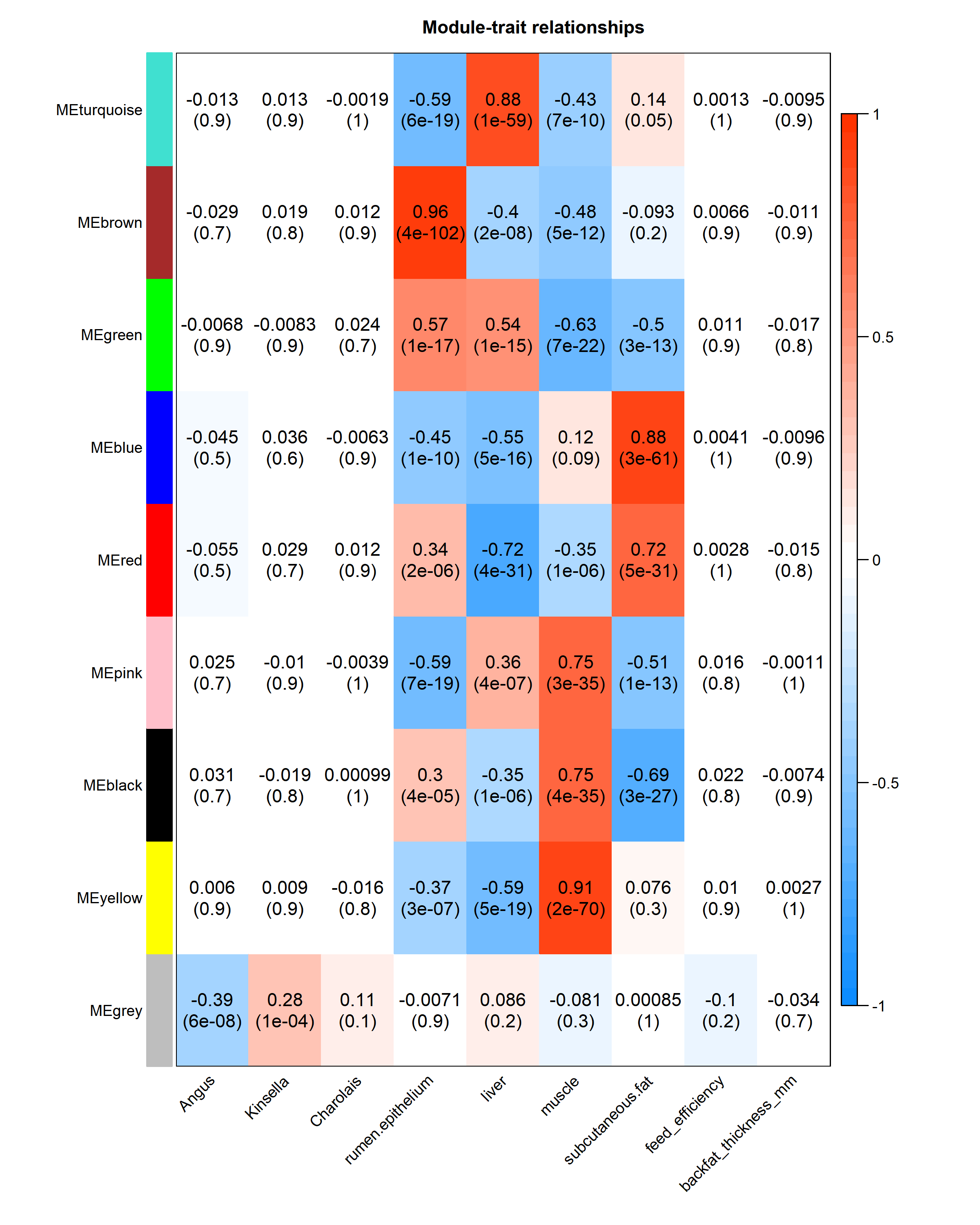

Supplement: Supplementary file 2 [file Data_Sheet_2.ZIP › 914848-Raw Data/figure/figure-3A.tiff]

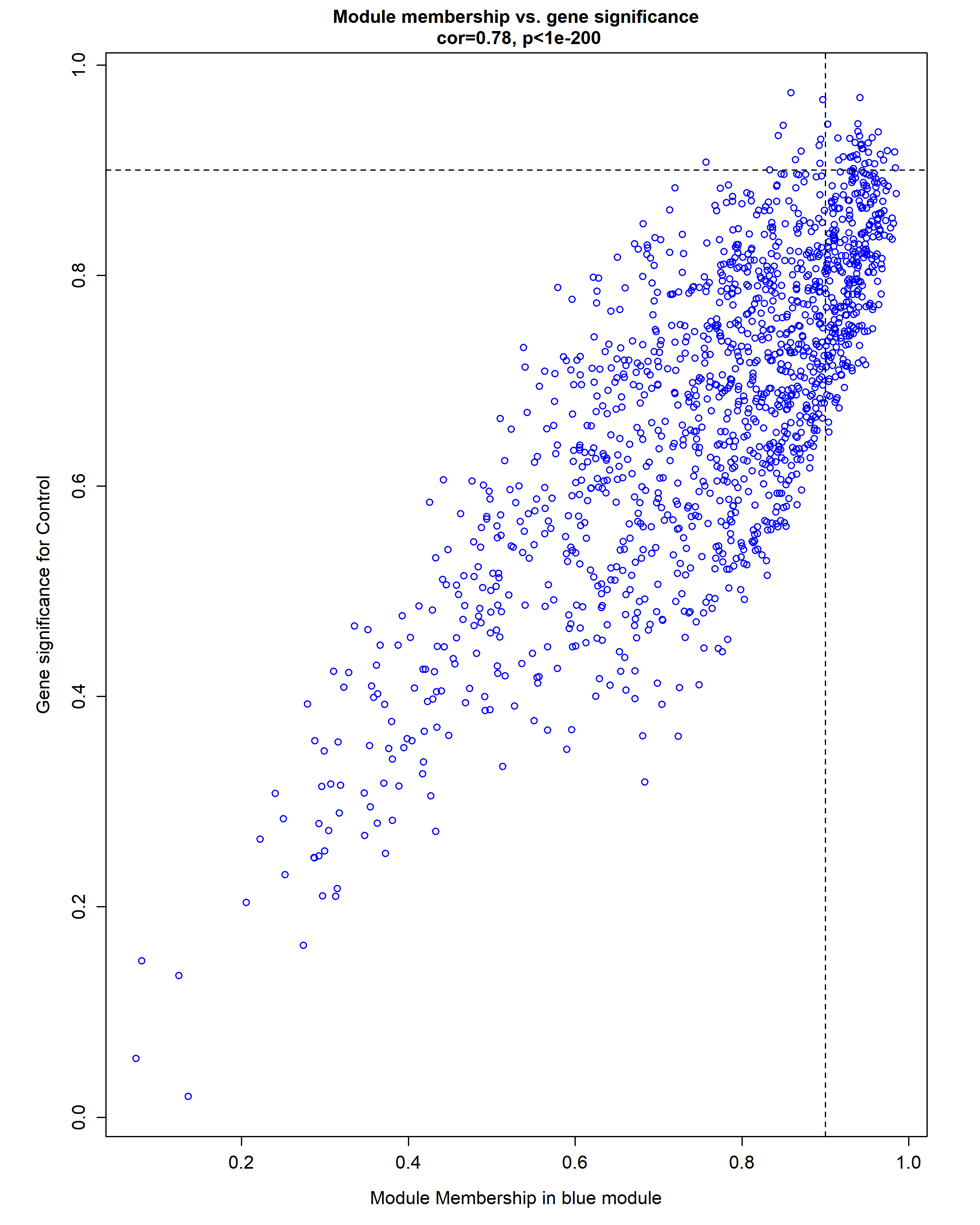

Supplement: Supplementary file 2 [file Data_Sheet_2.ZIP › 914848-Raw Data/figure/figure-3B.tiff]

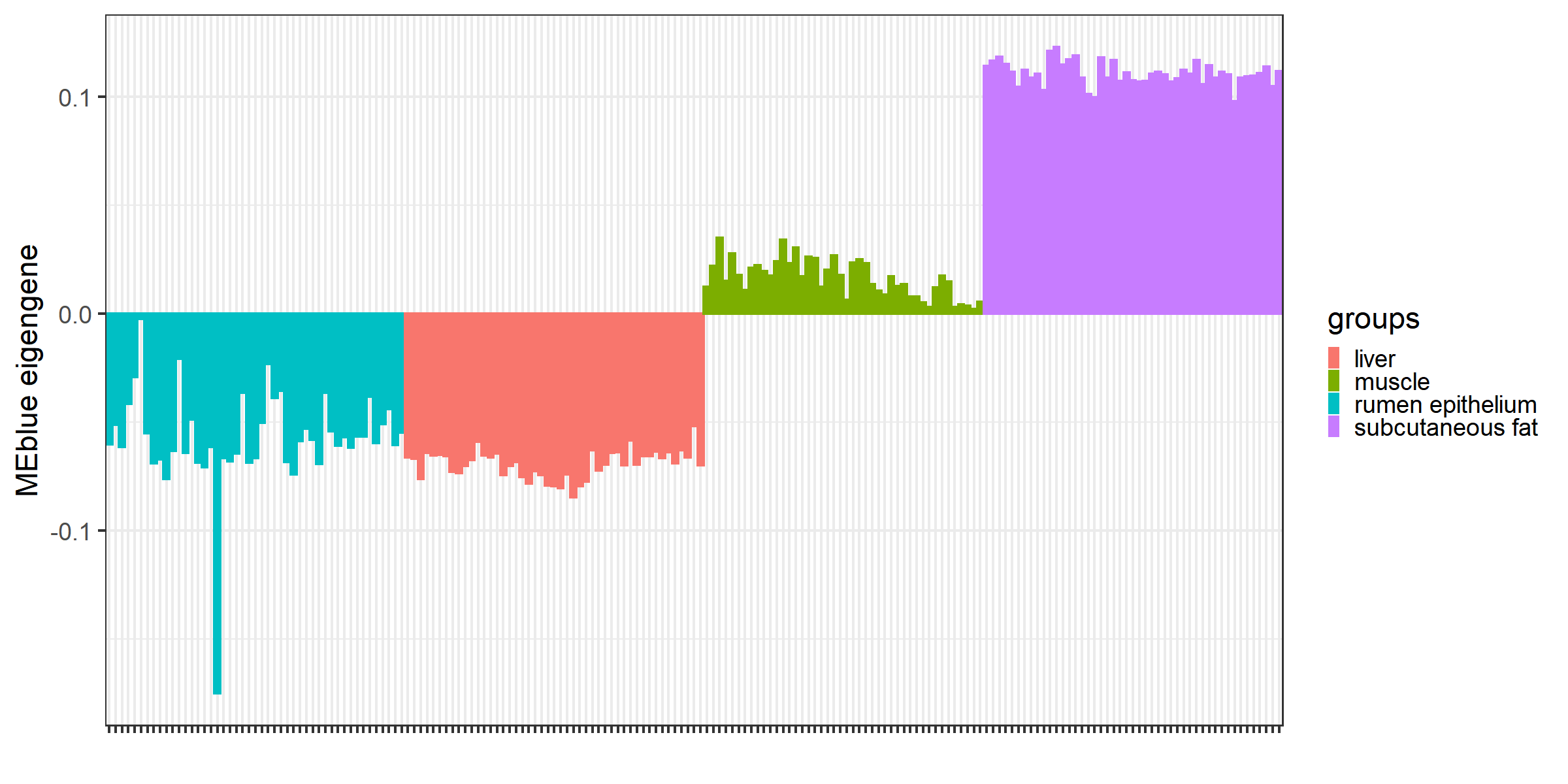

Supplement: Supplementary file 2 [file Data_Sheet_2.ZIP › 914848-Raw Data/figure/figure-3C.tiff]

# Biological Pathway

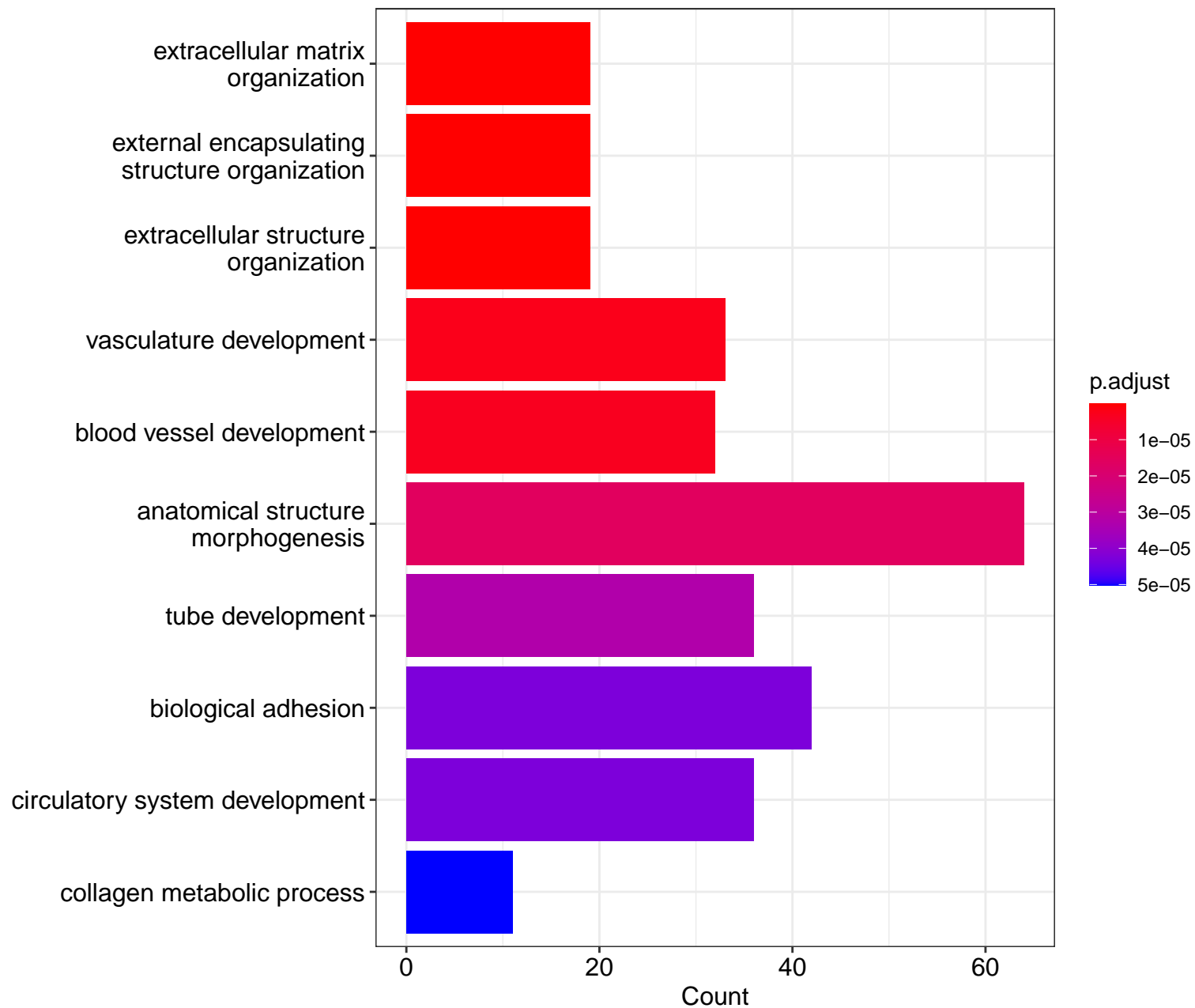

Supplement: Supplementary file 2 [file Data_Sheet_2.ZIP › 914848-Raw Data/figure/figure-4A.pdf]

# KEGG Pathway

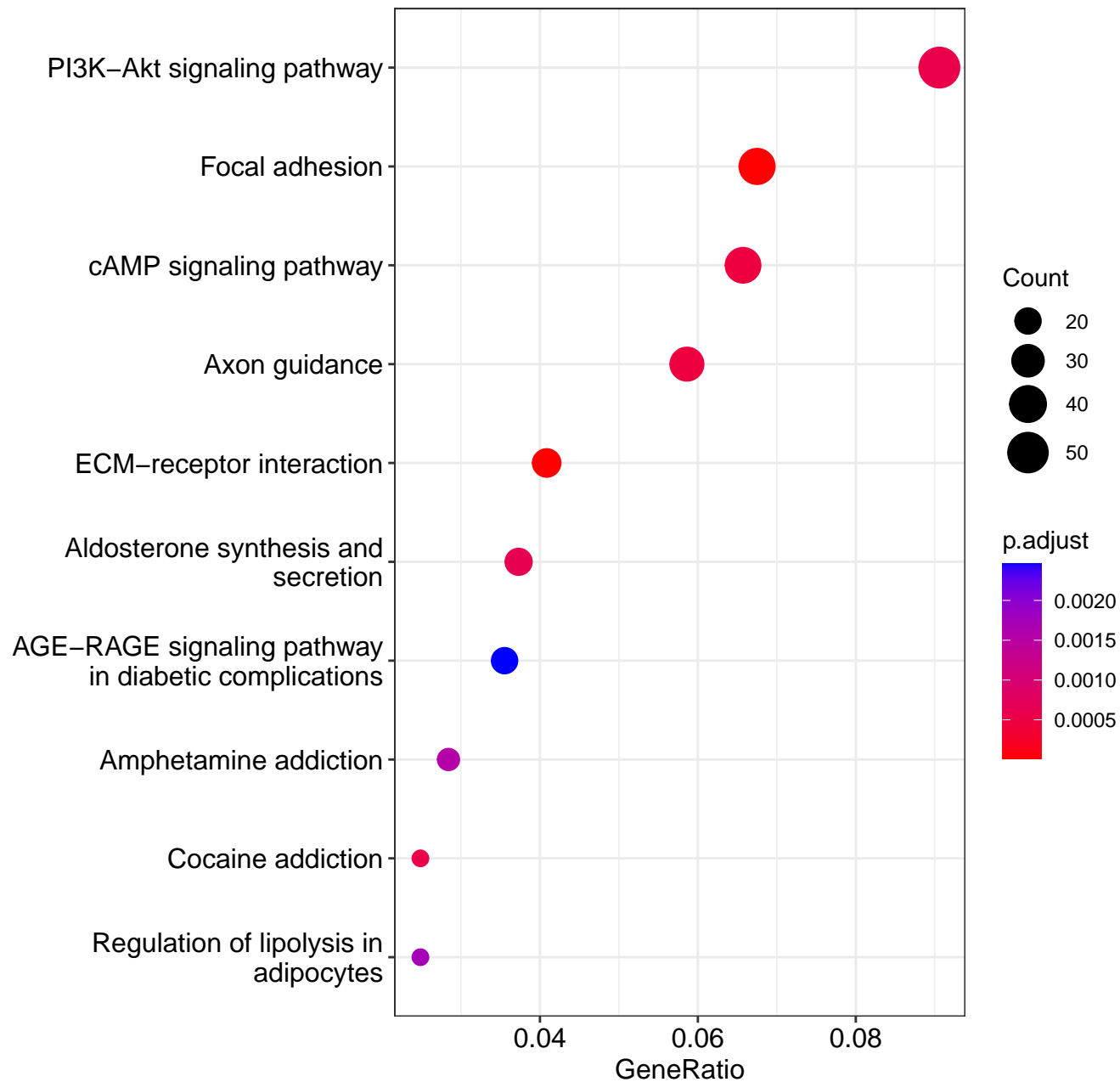

Supplement: Supplementary file 2 [file Data_Sheet_2.ZIP › 914848-Raw Data/figure/figure-4B.pdf]

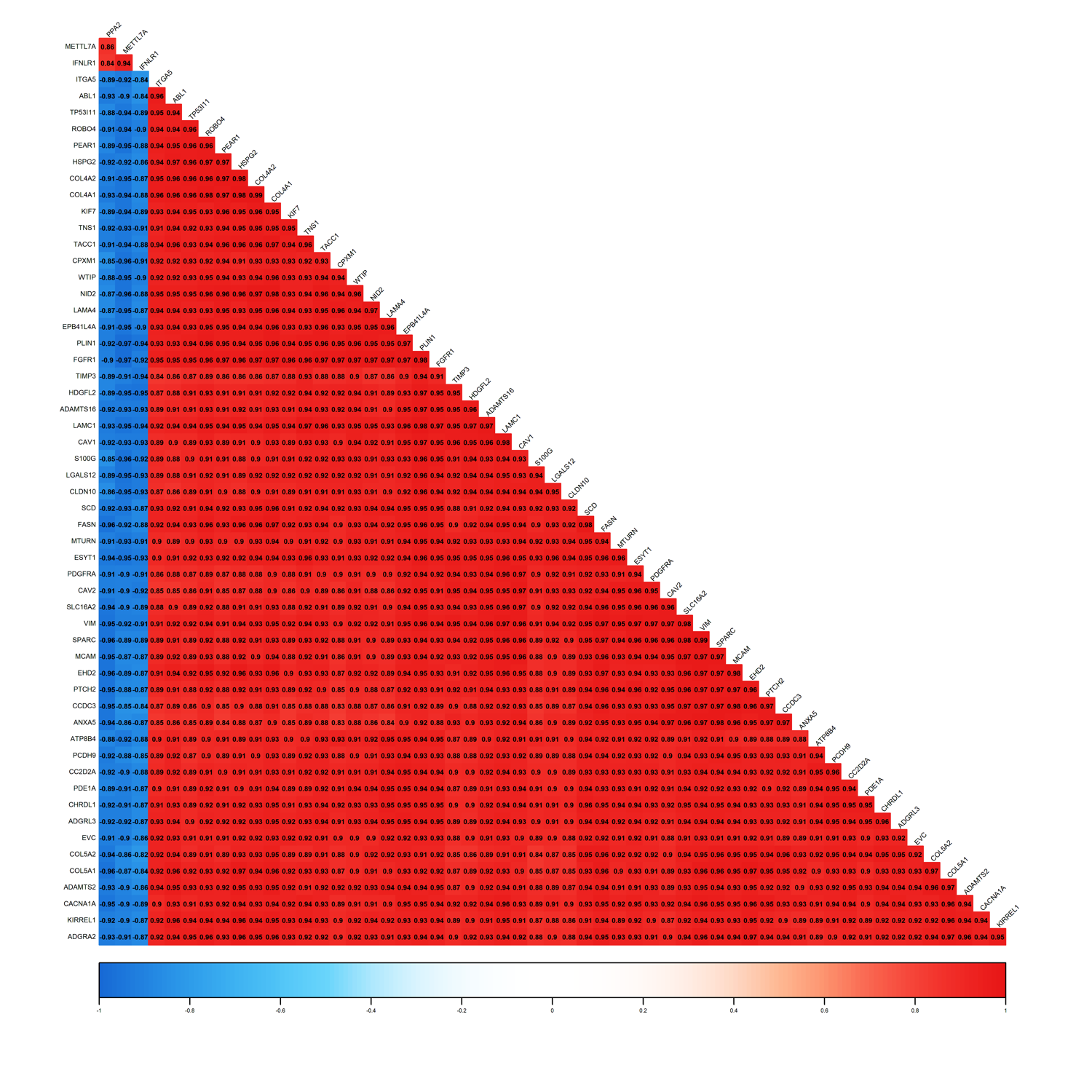

Supplement: Supplementary file 2 [file Data_Sheet_2.ZIP › 914848-Raw Data/figure/figure-5.png]

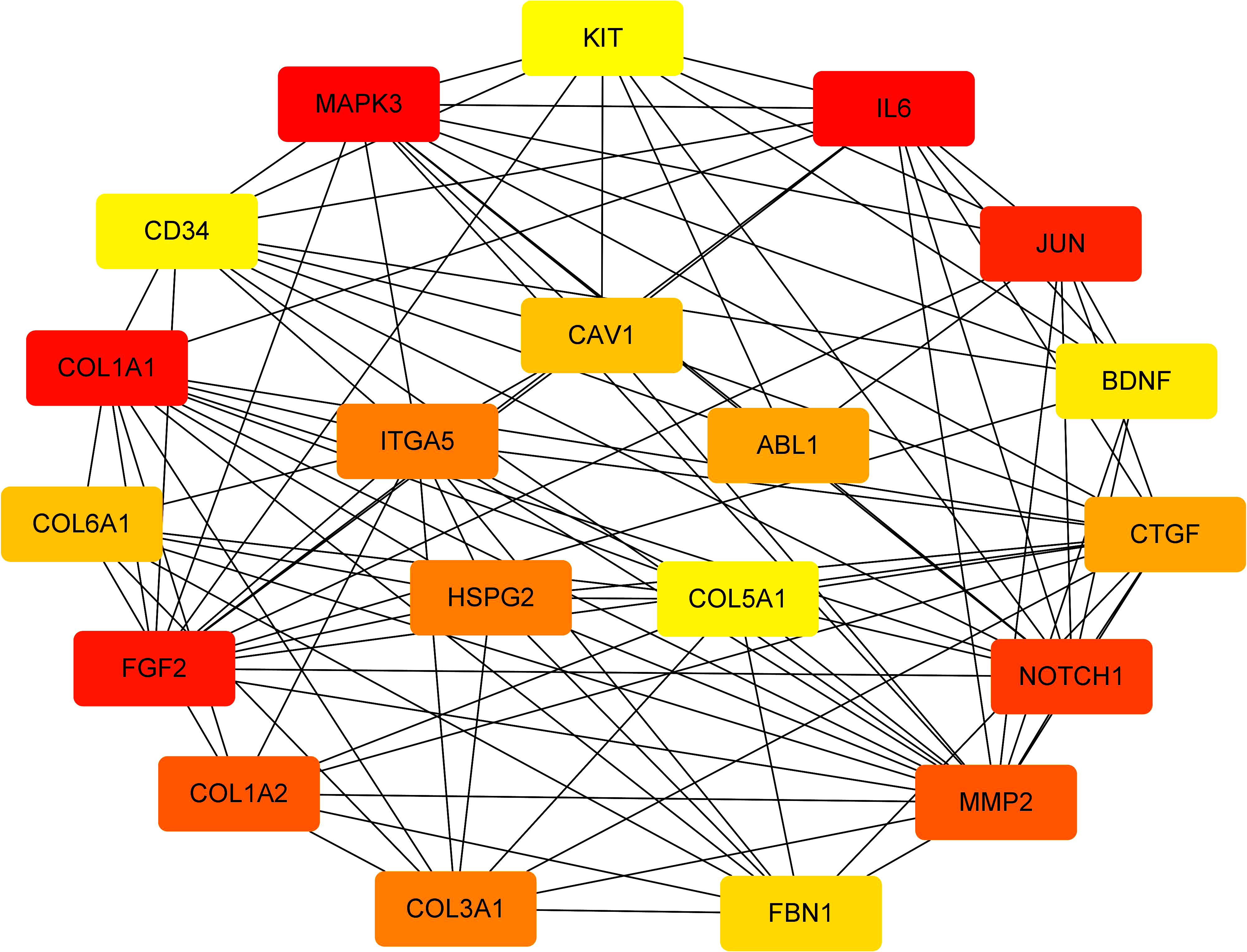

Supplement: Supplementary file 2 [file Data_Sheet_2.ZIP › 914848-Raw Data/figure/figure-6A.tif]

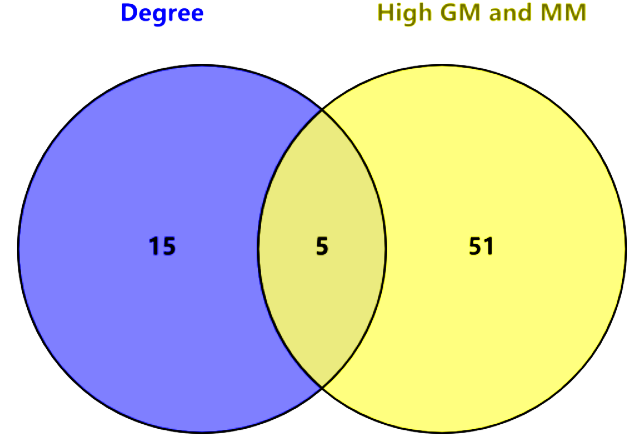

Supplement: Supplementary file 2 [file Data_Sheet_2.ZIP › 914848-Raw Data/figure/figure-6B.tif]

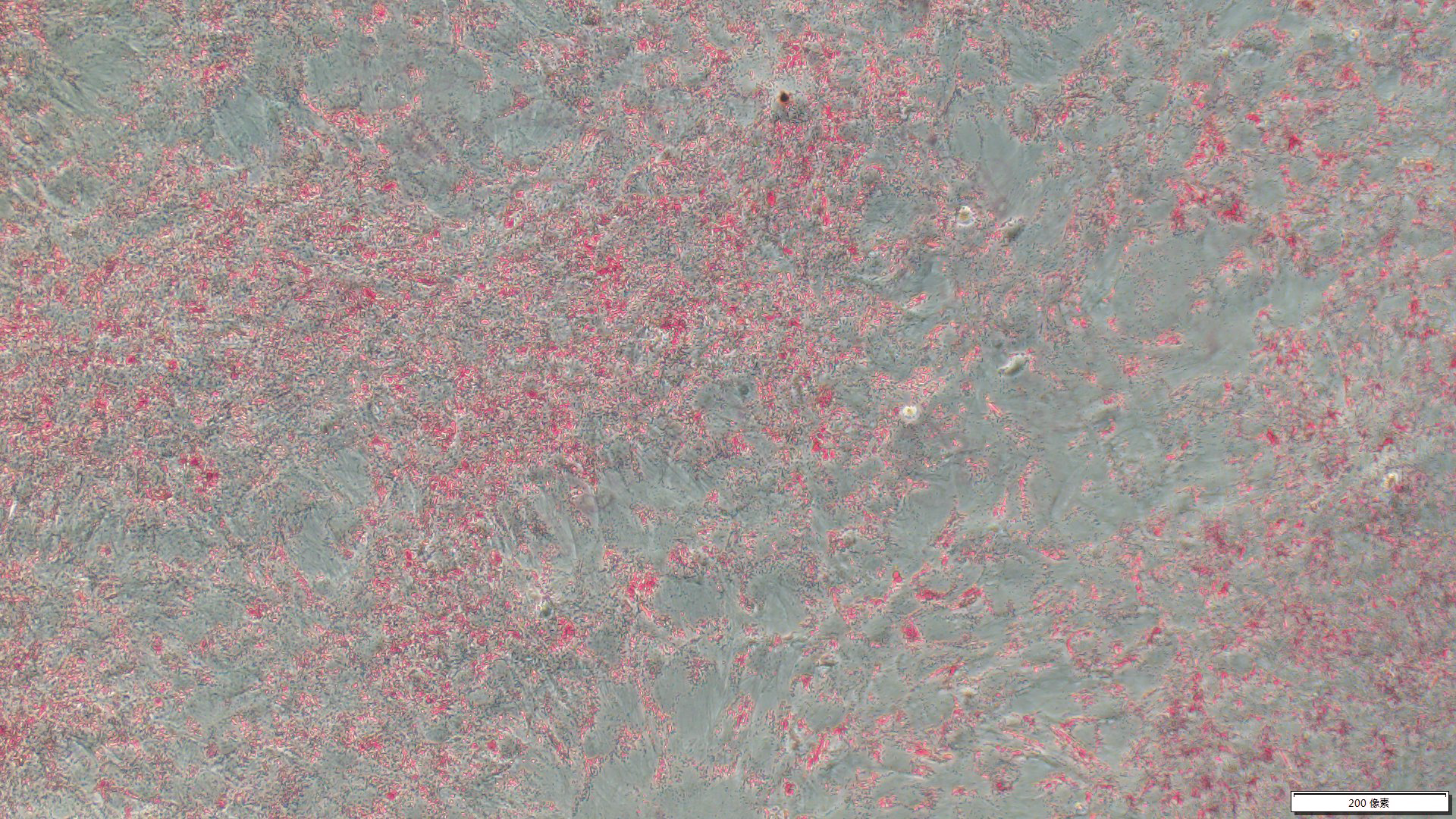

Supplement: Supplementary file 2 [file Data_Sheet_2.ZIP › 914848-Raw Data/figure/figure-7A1.tif]

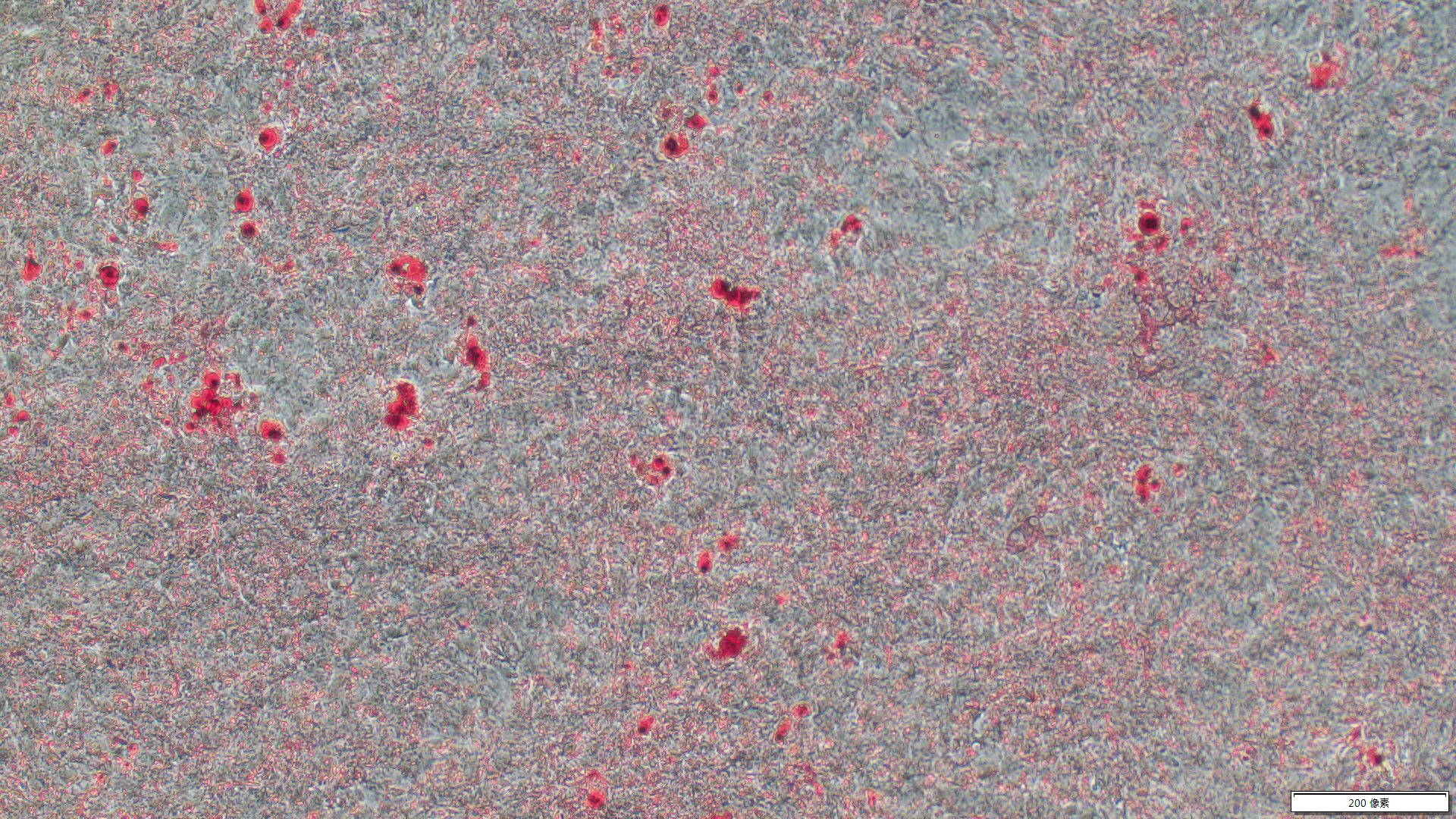

Supplement: Supplementary file 2 [file Data_Sheet_2.ZIP › 914848-Raw Data/figure/figure-7A2.tif]

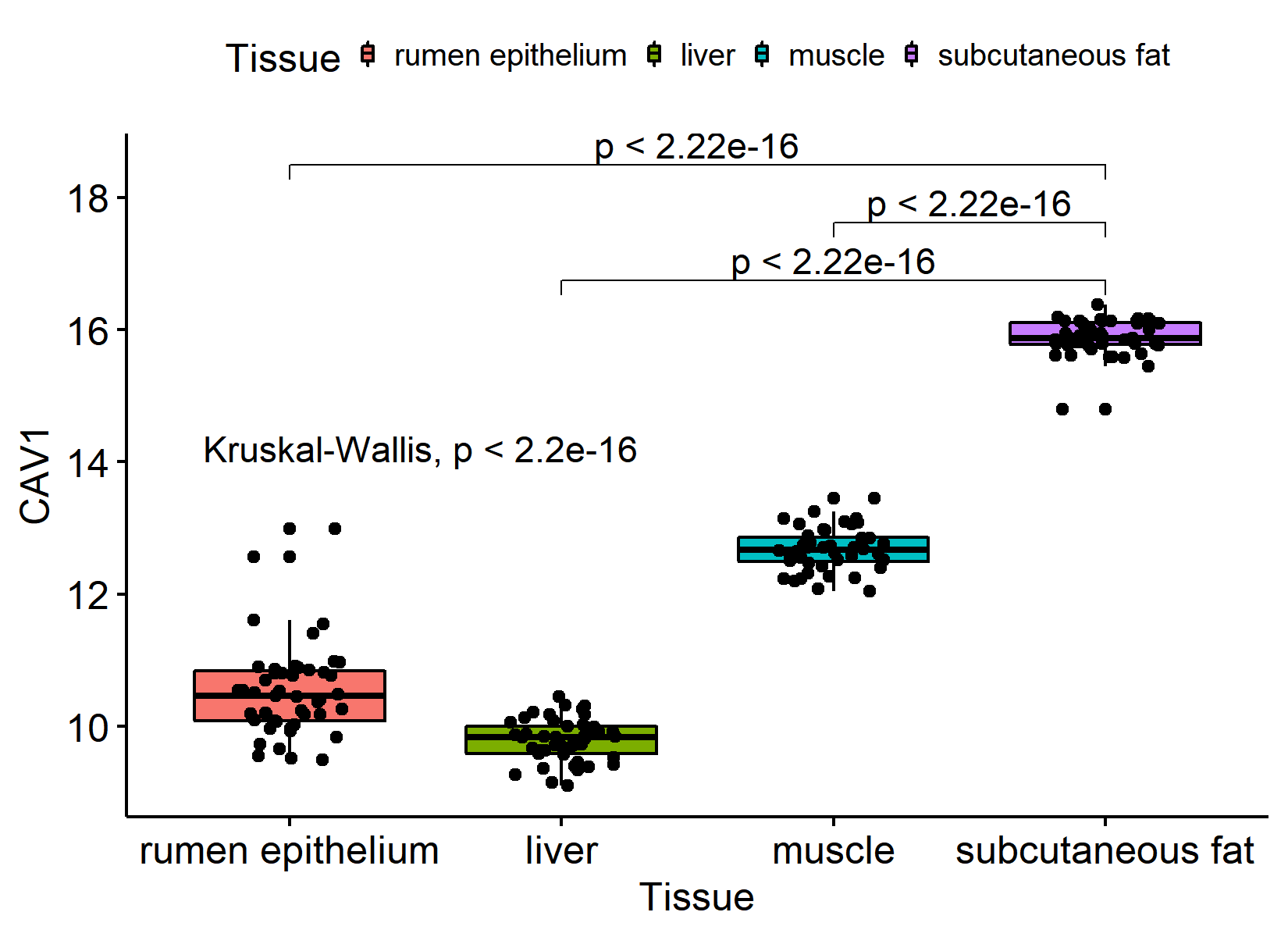

Supplement: Supplementary file 2 [file Data_Sheet_2.ZIP › 914848-Raw Data/figure/figure-8A.tiff]

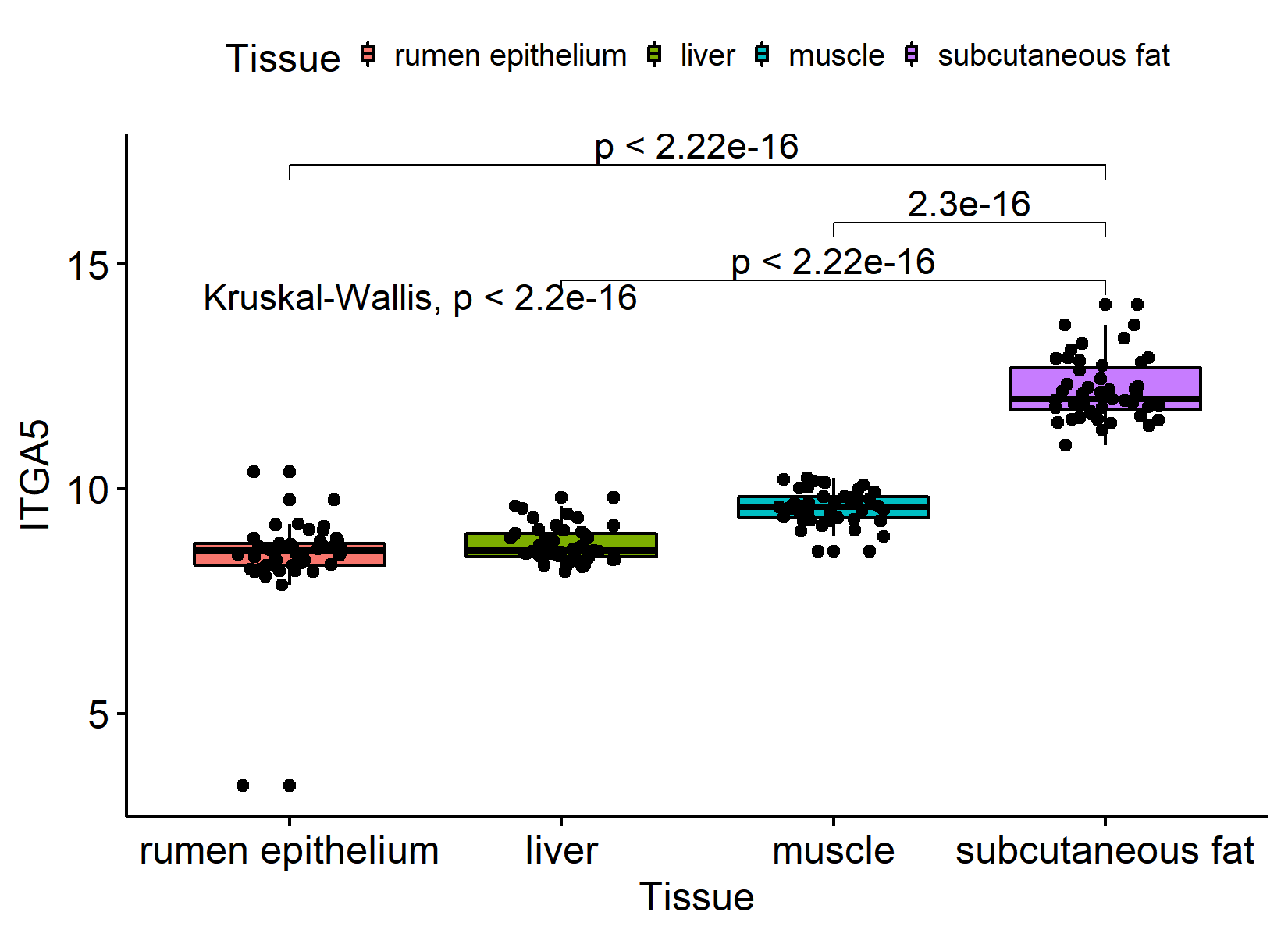

Supplement: Supplementary file 2 [file Data_Sheet_2.ZIP › 914848-Raw Data/figure/figure-8B.tiff]

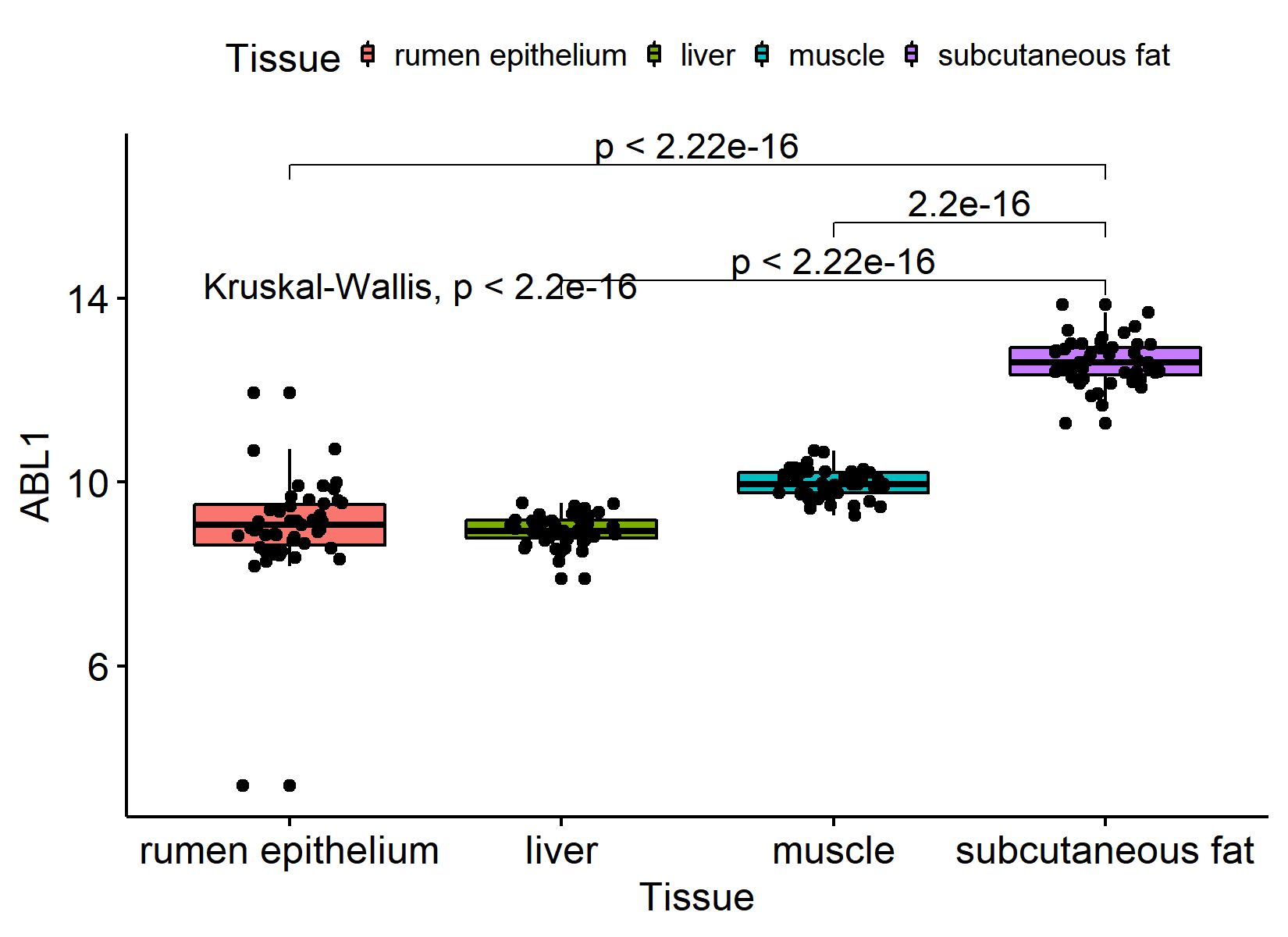

Supplement: Supplementary file 2 [file Data_Sheet_2.ZIP › 914848-Raw Data/figure/figure-8C.tiff]

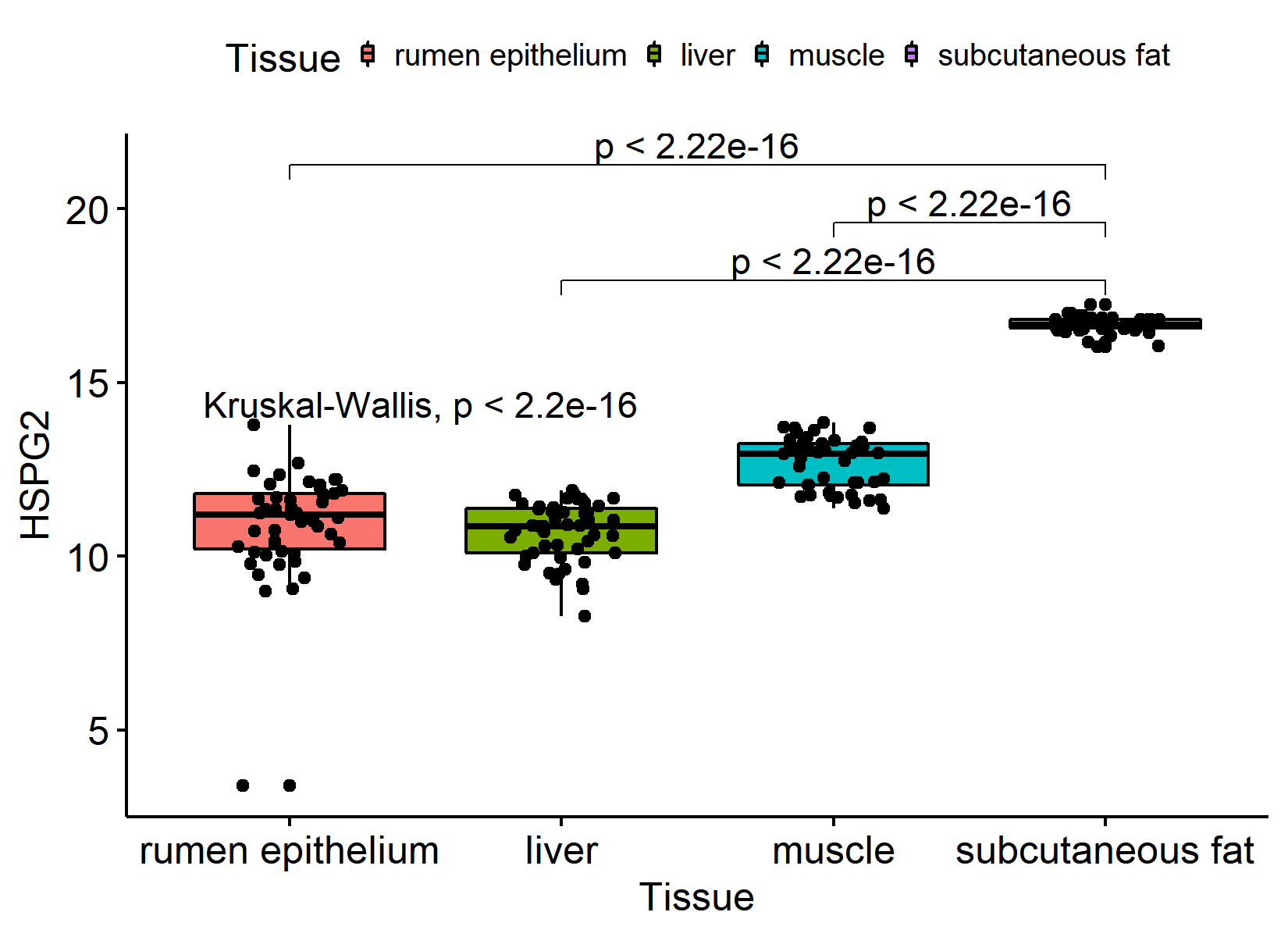

Supplement: Supplementary file 2 [file Data_Sheet_2.ZIP › 914848-Raw Data/figure/figure-8D.tiff]

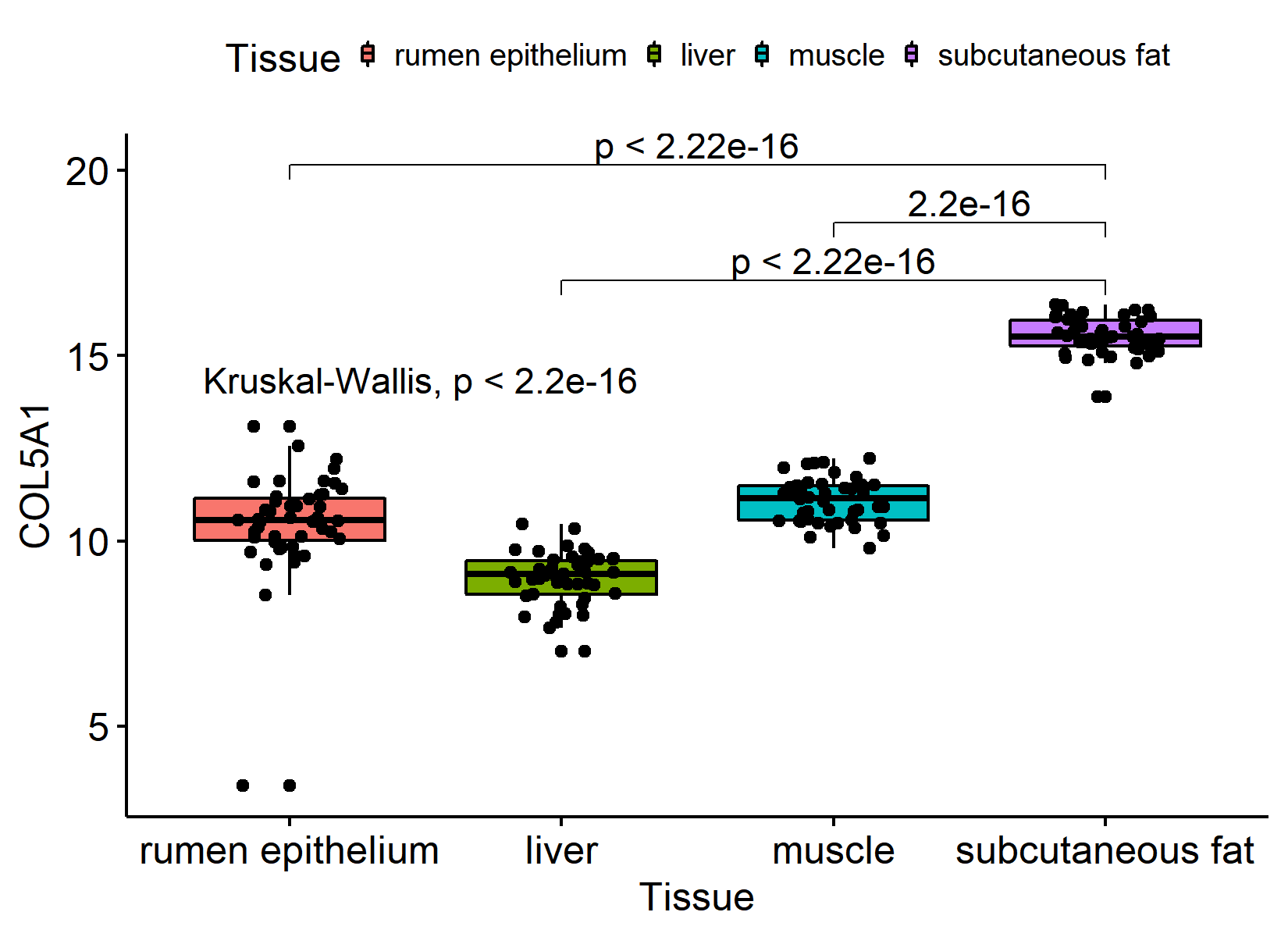

Supplement: Supplementary file 2 [file Data_Sheet_2.ZIP › 914848-Raw Data/figure/figure-8E.tiff]
